# Faithfulness-boost effect: Loyal teammate selection correlates with skill acquisition improvement in online games

**DOI:** 10.1371/journal.pone.0211014

**Published:** 2019-03-05

**Authors:** Gustavo Landfried, Diego Fernández Slezak, Esteban Mocskos

**Affiliations:** 1 Universidad de Buenos Aires, Facultad de Ciencias Exactas y Naturales, Departamento de Computación, Buenos Aires, Argentina; 2 CONICET-Universidad de Buenos Aires, Instituto de Investigación en Ciencias de la Computación (ICC), Buenos Aires, Argentina; 3 CONICET, Centro de Simulación Computacional p/Aplic Tecnológicas (CSC), Buenos Aires, Argentina; IBM Thomas J Watson Research Center, UNITED STATES

## Abstract

The problem of skill acquisition is ubiquitous and fundamental to life. Most tasks in modern society involve the cooperation with other subjects. Notwithstanding its fundamental importance, teammate selection is commonly overlooked when studying learning. We exploit the virtually infinite repository of human behavior available in Internet to study a relevant topic in anthropological science: how grouping strategies may affect learning. We analyze the impact of team play strategies in skill acquisition using a turn-based game where players can participate individually or in teams. We unveil a subtle but strong effect in skill acquisition based on the way teams are formed and maintained during time. *“Faithfulness-boost effect”* provides a skill boost during the first games that would only be acquired after thousands of games. The tendency to play games in teams is associated with a long-run skill improvement while playing loyally with the same teammate significantly accelerates short-run skill acquisition.

## Introduction

Skill is mainly acquired from individual experience. Humans, due to its social characteristic, also incorporate knowledge by learning from others. Social learning may affect the skill acquisition process expected from experience, and involve beneficial and risky alterations to subject abilities [1]. In this article, we exploit the virtually infinite repository of human behavior available in Internet to study a relevant topic in anthropological science: how grouping strategies may affect skill acquisition.

The study on expert decision-making grew out of research on master chess players [2–4]. When making decisions under uncertainty, experts rely on heuristics that generally lead to non-rational and suboptimal behavior [5, 6]. Individual experience has long been a major topic, studied as the main factor in performance improvement. Newell argued in 1981 that the generalized power law describes all of the practice data [7]. In recent years, some authors discuss that power law is limited to explain population learning curves and propose other functions to approximate individual learning curves [8]. However, the individual learning curves are more irregular than averaged learning curves and predicting large time scale performance based on small time scale events probed to be hard [9, 10]. Practice is an important learning factor but is not the only one. Other essential components should be taken into account in order to better understand the learning processes.

One of the factors that cause an alteration in expected learning curves is, for prosocial animals, the social learning ability. Social learning is defined as long-term changes in behavior caused by stimuli derived from observation of—or interaction with—other individuals [11, 12]. Our species was involved in a unique gene-culture coevolution that caused the emergence of our special social learning abilities: a costly cognitive machinery enabling efficient acquisition of complex traditions [13]. Humans learn things from others, improve and transmit them to the next generation, leading to a cultural accumulation that can not be developed by a single individual during her lifetime [14]. The ability to acquire behaviors based on the experience of others without having to build it by trial and error leads to a cumulative culture evolution, allowing humans populations to adapt rapidly to changes and new environments [1]. A degree of credulity is required for this process to work, and therefore social learners can acquire inappropriate information even in uniform and stable environments [15, 16]. Deciphering how to take advantage of social information while handling the inconveniences that arise from their use has become the main topic in the research on strategies of social learning [17, 18].

Many models of social learning strategies and their emerging population dynamics have been proposed. Researchers have identified several theoretical strategies [18, 19], which can be classified as: (a) those that specify the circumstances under which individuals copy others, and (b) those that identify from whom individuals learn. Recently, many studies of social learning have been conducted using different methods such as field observations [20], controlled laboratory experiments [19, 21–28] and field experiments [29–32]. Social learning now constitutes a major area of study within behavioral and evolutionary biology [33].

One difficulty inherent to all the mentioned methods is their reliance on small samples. With the advent of datasets from virtual communities, we set to study social learning in a massive data environment. We rely on a vast corpus (∼ 4.5 millions of games), capitalizing on a worldwide tendency of people to play multi-player online games and on the existence of servers that accumulate public data. This novel methodology seeks statistical emergent of potentially subtle effects, which may be detectable only with a remarkable number of observations and might remain undetected in a small sample sizes typical of laboratory studies [34]. Our study also incorporates the current capacity to analyze the value of skill acquisition with high accuracy. These results are obtained from a very unique experimental condition in which players engage in natural relationships, free to choose their teammates and opponents, and produce reliable outcomes which can be measured directly without hinge on indirect methods such as self-reported choice.

Online games have already been used as a model to study complex cooperative processes in social science [35, 36], neuroscience [34, 37], and computational social science [38–41]. Chess has been, by its complexity and clear rules, a privileged model for the study of learning and decision making. Massive chess data allowed the analysis of the influence of age, cohorts, gender and other features on learning [9, 10, 42–45].

Here we set to investigate the impact of team play strategies on skill acquisition in *Conquer Club*, an online multiplayer turn-based game. Unlike the individual game nature of chess, at *Conquer Club* (inspired by the board game RISK) a variable amount of players can take part in each game, playing individually or in teams (Section A in S1 File). In Conquer Club there is a strong incentive for collaboration: the results of the games are by teams. All the players of a team win or lose together. A player who is eliminated during a game can still end up winning if her teammates defeat the rest of the teams. Therefore, it is essential that teammates can coordinate their actions. In contrast to other platforms, there is no paid content, offering the same conditions for all players. There is no skill matching mechanism based on the probability of winning of the players. The platform has a “Join a Game” section, where all the players can see all the open games (Section A in S1 File). A typical Conquer Club game environment has four relevant elements: the current map with troops occupation in each region, the game status showing current round and a summary of players information, a public game chat, and a log of movements.

Researchers studying skill acquisition in chess rely on Elo, an estimator of skill used by the World Chess Federations [46, 47]. Elo can estimate a player’s skill, a hidden variable, by observing only her games outcomes. The model assumes that, in each game, the players exhibit performance, another hidden random variable related to the true skill value with some constant noise. The player who exhibits the greater performance is the winner. Under these assumptions, we can infer in each game who had the highest performance by observing the game outcome (win/lose). Moreover, based on the previous skill estimate we can compute the probability of an outcome, i.e. the probability that one player will have higher performance than her opponent. The skill estimator is updated according to the direction and magnitude of the surprise, i.e. the difference between the expected result (the prediction) and the observed result (the outcome of a game). We rely on *TrueSkill* [48], an extension of the Elo ranking system.

TrueSkill extends Elo through a Bayesian model. Firstly, TrueSkill uses a prior belief distribution, instead of a scalar, to represent the skill estimates. Since the initial skill value is unknown, the accepted procedure is to initialize all players with the same mean and a high variance. This allows the system to make big changes to the skill estimate early, and small changes after a series of consistent games have been played. As a result, TrueSkill can identify players’ skill through a few games. Secondly, TrueSkill adds a model of team performance, which allows the system to deal with any team assignment. The team’s performance assumption is only used to adopt the skill of individual players such that the team outcome can be best predicted based on the additive assumptions of the skills. Finally, TrueSkill uses a non-arbitrary update function, the posterior of the Bayesian model that could be computed by performing a marginalization over the factor graph [49] (See details at Methods section).

With our massive dataset, we can investigate the impact of team play strategies on individual skill acquisition that otherwise would not be possible to study.

## Results

### Law of practice

First, we study how players improve performance as they gain experience, i.e. the law of practice. We estimate the experience of each player by the number of games played. Skill is estimated according to the *TrueSkill* method [48]. The skill difference between opponents indicates with high precision the probability of winning (Fig A in S1 File). With two opponents (teams or individuals), the probability of winning when the other has the same skill is 1/2 and a difference of 4 tsp (TrueSkill points) increases the probability of winning to 2/3.

In our context, the learning curve is the skill progression as experience is acquired (i.e. the number of games played). As mentioned, population learning curves should follow a power law function [7],

$$Skill={Skill}_{0}\cdot {Experience}^{\alpha }$$ (1)

where *α* is the learning rate characteristic of the population, and *Skill*_0_ the population skill after the first game.

**Fig 1:**
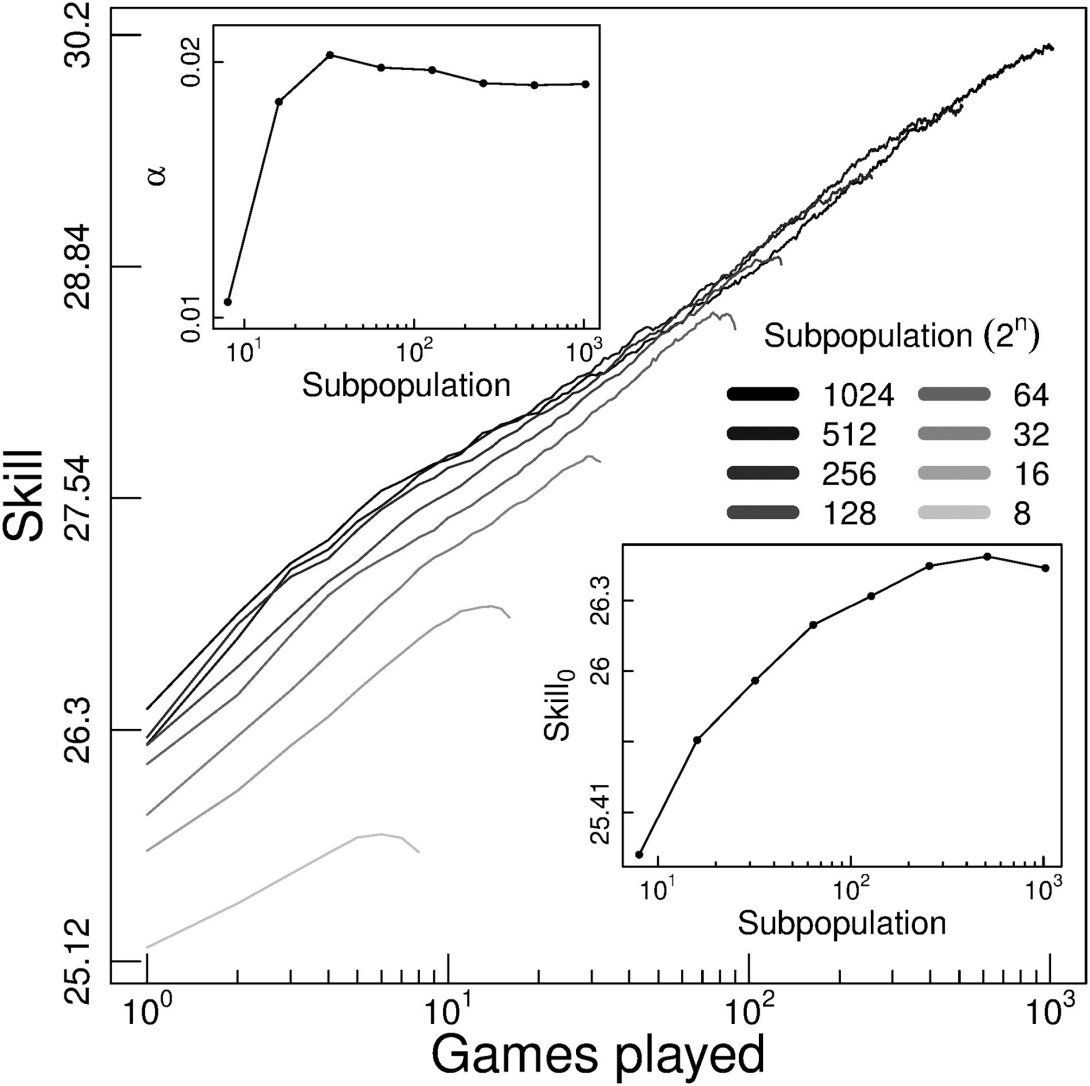
Law of practice. Log-log learning curve of the subpopulation of players with different total activity. Each learning curve shows the skill of the first 2^*n*^ games played of the subpopulation with at least 2^*n*^ games played and less than 2^*n*+1^. Subplots show the parameterized values (i.e. *α* and *Skill*_0_) of each learning curve following Eq 1.

To analyze the law of practice, we split players according to their total activity: (1) players with at least 8 games and less than 16, (2) players with at least 16 games and less than 32, and so on. Thus, we fit these parameters to each set of players in these subpopulation activity categories. In concordance to the law of practice, we observe a linear dependency in the log-log learning curves in all the population segmentation (Fig 1).

Learning curves have a dependency on players who churn out, showing lower skill for subpopulations with lower total activity. However, the learning rate (*α*) remains stable for subpopulations with at least 32 games played (upper right inlet in Fig 1). The difference between them causes little variation in long-term skill acquisition, providing less than 0.24 tsp after 1000 games played. The initial subpopulation skill (*Skill*_0_) is also affected by churning out (bottom left inlet in Fig 1). Nevertheless, all subpopulations with at least 64 games played, do not have a significantly different initial skill (Wilcoxon rank-sum test at Table A in S1 File).

Therefore, all cohorts with at least 64 games played have almost equivalent learning curves. They are what we call the “learning curve expected by experience”. This baseline learning can be altered by many factors. For example, the commitment to finish the games is undoubtedly a relevant factor in the process of skill acquisition. Indeed, players who always finish their games have a higher learning curve than the rest of the population, around 0.5 tsp (Fig C in S1 File).

### Social learning

Social learning is essential to pro-social animals. We hypothesize that the learning curve expected by experience could be altered by different grouping behaviors. To study it, we analyze players’ behavior in team selection.

In the game platform, users can choose between playing individually or in teams. We define players’ *team-oriented behavior* (TOB) as the number of team games played divided by the total number of games played:

$$Team-oriented\;behavior=\frac{Team\;games\;played}{Games\;played}$$ (2)

To evaluate the influence of TOB on learning curves we split the population into strong, medium and weak TOB (i.e. 0.8 < *TOB* ≤ 1, 0.4 < *TOB* ≤ 0.6, and 0 < *TOB* ≤ 0.2, respectively). Hereinafter, we excluded players with less than four team games played.

**Fig 2:**
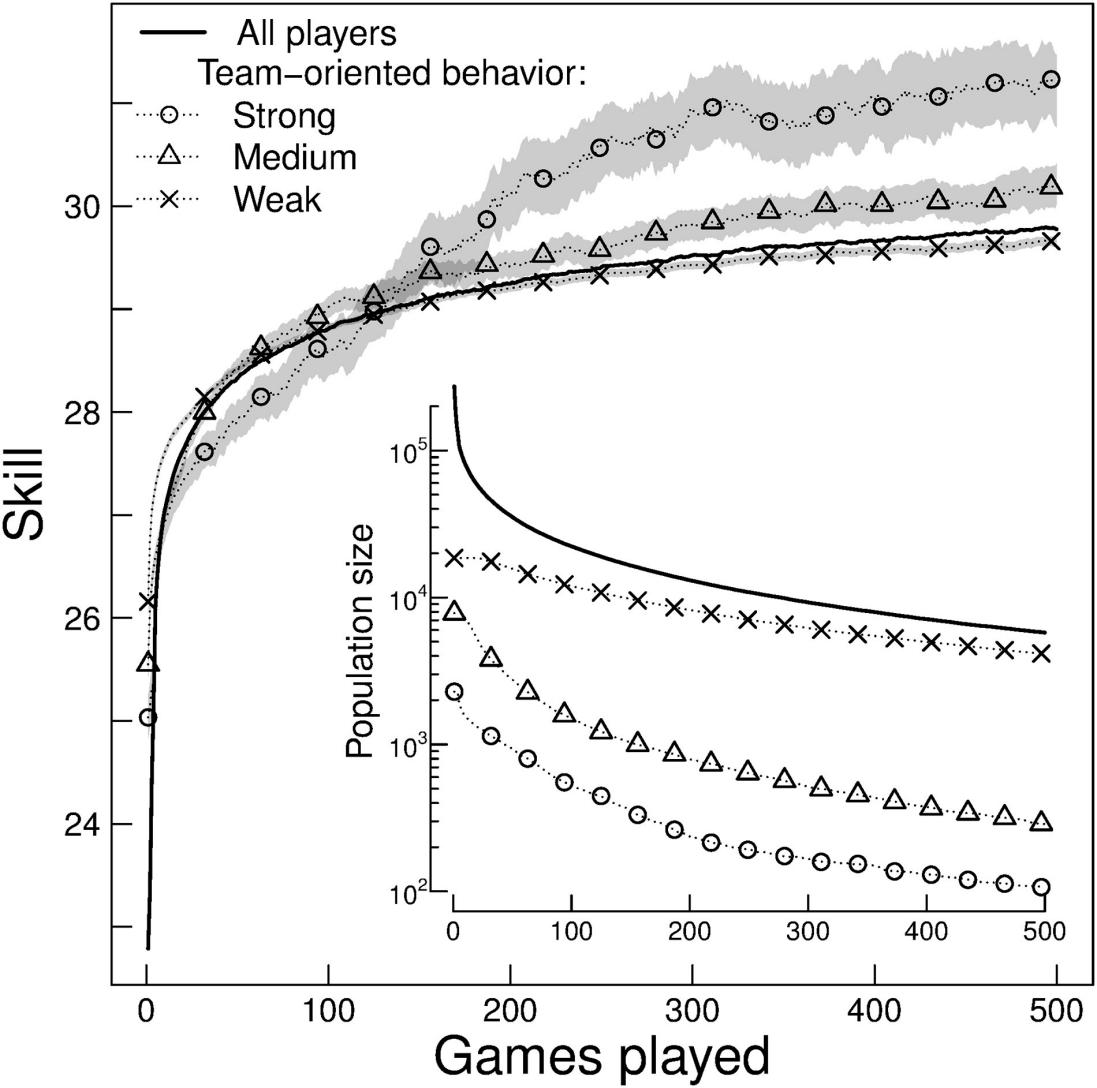
Social learning. The learning curve for strong, medium and weak team-oriented behavior. The band represents 95% Wilcoxon rank-sum confident interval, and the middle line represents the pseudomedian. As a reference, we show the learning curve of the whole population. Results are analogous to those obtained with mean and 95% t-test confident interval.

In the long-run, between 200 and 500 games of experience, the learning curves are ordered according to their TOB level, exhibiting higher skill level for populations with higher TOB (Fig 2). The strong team-oriented players evince after 250 games played, a significantly higher final skill compared to medium and weak TOB (Wilcoxon rank-sum test, *p* < 1 × 10^−4^). In this interval strong and medium TOB population are distanced by about 1 tsp. A more team-oriented behavior has, in the long-run, higher skill value even compared with players without team games (i.e TOB = 0, Fig D in S1 File).

### Faithfulness-boost effect

Players can choose between playing with the same teammate or selecting different players in each game. We hypothesize that a loyal behavior may affect learning (increase or decrease the rate of skill acquisition) when playing in teams. If we look at how many recurrent players each player has, we find that most recurrent players are teammates instead of opponents. Thus, we focus our analysis only on the loyalty of teammates as loyalty in opponents is not present in our database. We define players’ *loyalty* as the proportion of times played with the most recurrent teammate divided by the number of team games played:

$$Loyalty=\frac{Maximum\;of\;games\;played\;with\;a\;partner}{Team\;games\;played}$$ (3)

To evaluate the influence of loyalty over learning, we examine players’ skill evolution in strong TOB based on their loyalty value. We define a player as *loyal* when *loyalty* > 0.5, and a player as *casual* when *loyalty* ≤ 0.2.

**Fig 3:**
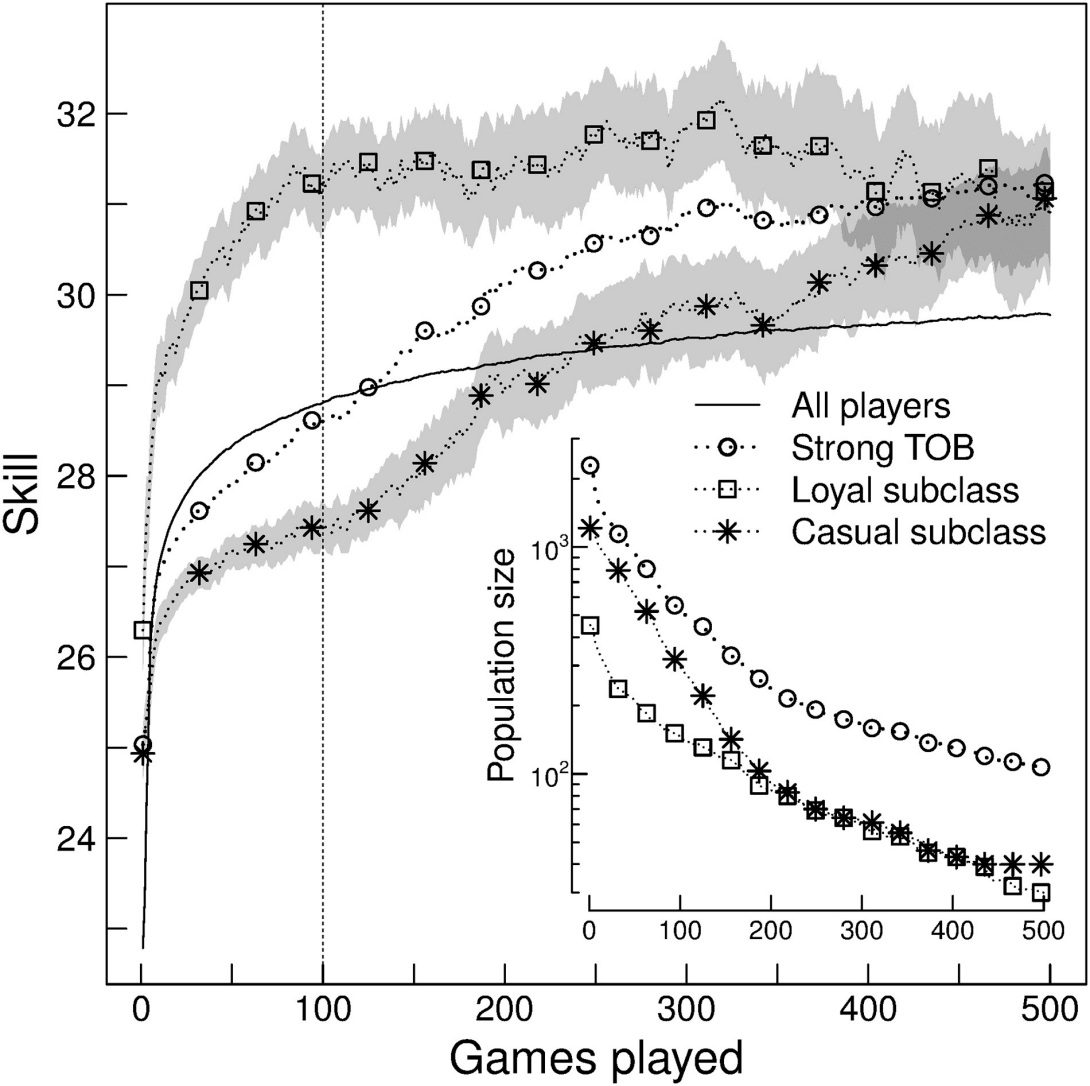
Loyalty influence over strong TOB learning curve. Learning curves of the loyal and casual subclasses of strong TOB. The band represents 95% Wilcoxon rank-sum confident interval, and the middle line represents the pseudomedian. As a reference, we show the learning curve of the whole population and the strong TOB. Results are analogous to those obtained with mean and 95% t-test confident interval. The vertical line at 100 of games played indicates the analysis performed in Fig 4.

If we compare the learning curves of loyal and casual players, we obtain a substantial separation between them at the first games of experience. Loyal players show an increment in the median skill of approximately 4 tsp over casual players (Fig 3). The skill distribution at each point of the learning curve is significantly different until 386 games played (Wilcoxon rank-sum test *p* < 0.01). An analogous behavior between loyal and casual subclasses is found for medium and weak TOB, less intense as they are less team-oriented (Fig E in S1 File).

**Fig 4:**
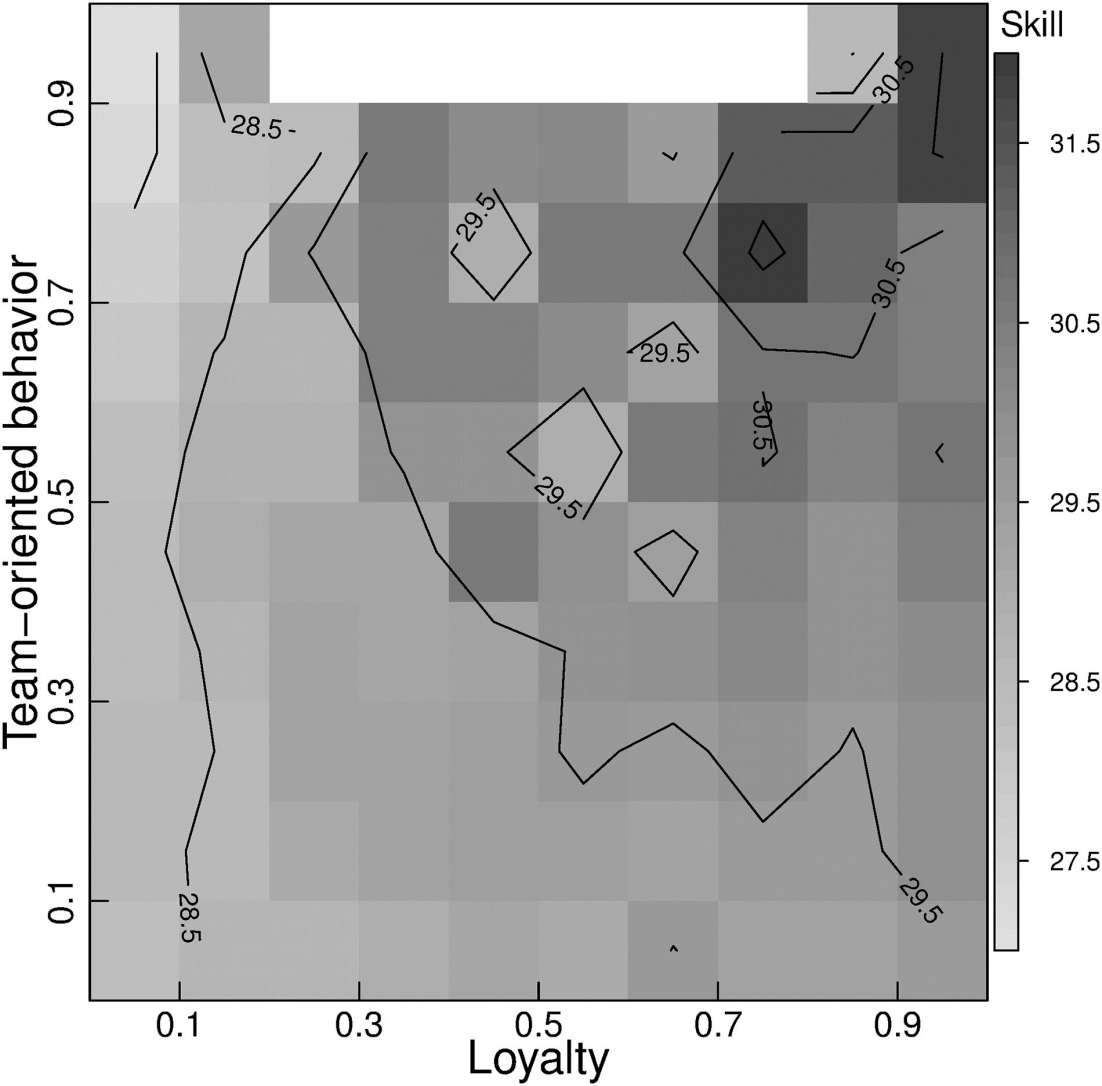
Skill interaction between loyalty and TOB for all players. The role of experience was isolated by taken the skill of players at the same point of experience. All players have 100 of games played. The average skill of each bin is reported by the gray-scale. Contour lines are shown. Empty bins have less than five players.

To study the interaction between TOB and loyalty on skill acquisition, we fix the number of games played to 100. By isolating this interaction (without the interference of experience) we find that an increase in loyalty always implies an increase in skill, more prominent for higher TOB values (Fig 4). Conversely, increasing TOB values shows a decrease in skill for low levels of loyalty, and only implies an increase in skill for high levels of loyalty. The skill difference from the minimum to maximum is greater than 4.5 tsp.

The interaction between loyalty and TOB can be summarized by *Loyalty* · *TOB* = *faithfulness* defined as,

$$Faithfulness=\frac{Maximum\;of\;games\;played\;with\;a\;partner}{Games\;Played}$$ (4)

which is simply the proportion of times played with the most repeating teammate over all the games played.

To measure the influence over skill acquisition of loyalty, TOB and faithfulness, we build a linear model solved by least squares. The correlation between variable loyalty and TOB is low (0.11), and the Variance Inflation Factor is nil (1.01), suggesting no evidence of collinearity.

$${\text{skill}}_{i}∼\beta_{1}{\text{loyalty}}_{i}+\beta_{2}{\text{TOB}}_{i}+\beta_{3}{\text{faithfulness}}_{i}$$ (5)

At 100 games played, loyalty has a significant positive slope, TOB has a significant negative one, while faithfulness has a significant steep positive slope (Table 1). Faithfulness is strong enough to reverse the negative TOB contribution over skill to a positive one when *loyalty* > 0.27. The *faithfulness-boost effect* is around 3.7 tsp, which generates a skill difference between players with the same experience extremely relevant in terms of probability of winning.

**Table 1:** Influence over skill acquisition of loyalty, TOB and faithfulness (linear model). We report the estimated slope value, their standard deviation and their significance difference with respect to a zero slope. All players have 100 games of experience.

	Estimate	Std. Error	t value	Pr(> |*t*|)
Intercept	28.5707	0.0405	705.59	*p* < 2*e*^−16^
Loyalty	0.7594	0.0972	7.82	*p* < 2*e*^−14^
Team-oriented	-1.0042	0.1088	-9.23	*p* < 2*e*^−16^
Faithfulness	3.7077	0.2611	14.20	*p* < 2*e*^−16^

We repeat this procedure for players with the same experience, starting from 100 to 1300 games, by 100 games played (Fig F in S1 File). The *faithfulness-boost effect* remains significant until 400 games played, always above 3 tsp. Starting at 500 played games, faithfulness ceases to be significant but TOB slope reverses its contribution to a significantly positive one. Loyalty has a significant positive effect at any level of experience (≥ 100 games played). Although the interaction effect of the linear model (faithfulness) is no longer significant from 500 games of experience onwards, the point of maximum skill is always achieved by maximizing both loyalty and team-oriented behavior. The magnitude of this contribution is always relevant in terms of winning probability, with more than 2 tsp.

In order to integrate all the partial observations made up to here, we perform one overall model fitted to all data including experience, loyalty, TOB as the predictors, and individual player as a random effect (Table 2). We choose a linear mixed model because the relationship between the experience and skill is linear on a log-log scale (Eq 1). The dependent variable, players’ skill, was transformed to a logarithmic scale,

$${\text{log}}_{10}(skill)={\text{log}}_{10}(experience)+loyalty+TOB+individual\;player+\varepsilon$$

**Table 2:** Linear mixed model for one overall model fitted to all data between 10 and 500 games played of individual experience. At column normalized estimates (Norm.Est.) we transform the estimators, in logarithmic scale, to their normalized value, (e.g. Intercept = 10^1.415^, exp = 10^1.415+ 0.016^ − 10^1.415^). Method: REML converged. Number of groups: 65335. Max Group size: 491. Mean group size: 99.5.

	Estimate	Norm.Est.	t value	[0.005	0.995]
Intercept	1.415	26.00	5966.264	1.414	1.415
exp	0.016	0.98	225.628	0.016	0.017
loyal	-0.007	-0.42	-28.846	-0.008	-0.006
tob	-0.044	-2.51	-91.773	-0.045	-0.043
(faithful) loyal:tob	0.090	5.99	91.801	0.088	0.093
exp:tob	0.016	0.98	70.829	0.015	0.016
exp:loyal	0.004	0.24	29.678	0.003	0.004
exp:loyal:tob	-0.026	-1.51	-56.129	-0.027	-0.025
Group Var	0.002				

The dataset used contains the values of all the players for each game between 10 and 500 games played of individual experience. The collinearity between variables is nil in terms of Variance Inflation Factor computed from a linear model solved by least squares (i.e. all VIF less than 1.5). Therefore, we can fit the linear model without incurring in artificial results.

This overall model confirms the observations already introduced. Experience is the main predictor in terms of their level of significance, has the most significant slope. Initially, loyalty only has a positive effect in contexts of team-oriented behavior (loyal:tob). Be loyal without a team-oriented behavior has a very marginal effect (loyal). Without a stable teammate, playing team games results in a bad plan (tob). However, as more experience is gained, while the boost-effect provided by faithfulness interaction loses strength (exp:loyal:tob), the loyalty (exp:loyal) and team-oriented behavior effect (exp:tob) reverses their contribution to a positive one. Due to this dynamic, the point of maximum skill is always reached with both maximum team-oriented behavior and loyalty (i.e. maximum faithfulness).

## Discussion

Traditionally, learning is modeled as a function of experience. In this article, we focus on how the learning curve expected from practice could be altered by different grouping strategies. We exploit the virtually infinite repository of human behavior available on the Internet to study a relevant topic in anthropological science: how grouping strategies may affect skill acquisition. Our method is based on massive data which enabled conducting a longitudinal study with very high precision to detect subtle changes.

We analyze learning in the context of competing players, such as chess or RISK. In this types of games, learning is measured in terms of the probability that a player beats others. Unlike tasks in which it can be determined the absolute amount of errors that an individual makes when solving it, in competitive games the probability of winning is a relative property that depends on the learning level of opponents. Learning curves arising from tasks in which the skill is measured in relative terms are more volatile than those measured in absolute terms. Thus, individual learning curves of competing players are sometimes hard to fit [9, 10].

Regardless, the individual learning curves are more irregular than averaged learning curves and the variation among them must be explained. We hypothesized that social learning would expose a second-order effect in skill acquisition. According to social learning theories, players have two options for learning: i) discover for themselves the keys to better play; or ii) imitate the strategy of others available in her network. We rely on Conquer Club, an online game that—in contrast to chess—may also be played in teams. In Conquer Club both opponents and teammates are observable and, in consequence, they could be seen as models to imitate. However, we focus our analysis only on the loyalty of teammates as loyalty in opponents is not present in our database.

For instance, a simple social learning strategy consists of copying the majority of other available models, which is known as *frequency-based strategy*. Another social learning strategy is copying the most successful available model, named as the *payoff-based strategy*. As far as we know, no social learning strategy has been proposed which takes into account different grouping strategies. We found that grouping strategies affect significantly how the skill is acquired.

As in other species, it has been studied in ancient anthropology that homo-sapiens success relies on group formation [13, 50]. We explored if this behavior affects skill acquisition using a controlled environment, i.e. Conquer Club. The decision on playing individually or in teams (i.e. the team-oriented behavior) is associated with a long-run skill improvement. We found that tendency of playing in groups (the number of played team games divided by the total number of played games) improves significantly the skill level achieved.

Mathematical models show that relations between groups by individual migrants can be a risk factor for social learning when environments in which groups live differs [51]. We studied a reductionist version of migration among groups by analyzing how players repeat teammates, that leads to a intra-group stability (i.e. loyalty). Following environment change risk theory, we claim that there are no environmental modifications in Conquer Club and therefore inter-group mobility is not a source for the spread of maladaptive ideas. On the contrary, migration would imply the access to different groups and thus learn from different players an increase in skills by learning from a larger variety of teammates. In this sense, we tested the hypothesis that migration is beneficial for improving skill acquisition. However, we found the opposite.

First, we check the law of practice in our dataset. We found the empirical shape of what we call the “learning curve expected by experience”. Taking it as a baseline, we quantify to what extent different grouping behaviors alter the skill acquisition expected by experience. We found that a team-oriented behavior (the proportion of the played team games and the total played games) is related to a significant improvement of skill level achieved in the long-run. A tendency of an intra-group stability (loyalty, i.e. the number of times played with the recurrent teammate divided by the team games played) is associated with a rapid skill improvement in the short run. The combination of these two features contains a positive effect that may be exploited by learners. This *faithfulness-boost effect* provides a skill boost that would be acquired, through experience, only after thousands of games of practice.

We claim that the current skill of the potential partner may be ignored. There are no side effects derived from the skill heterogeneity between teammates. The winning probability of a team is independent of the difference between teammates (Fig G in S1 File). It is also important to point out that being part of a team with a low probability of winning does not mean losing the skill. Partnering with a lower-skill teammate will effectively entail a decrease in the probability of winning but not necessarily imply a decrease in skill. If collaboration is strong, both players will benefit from skill acquisition.

The evidence leaves important open questions that may have practical implications for planning training strategies. Our hypothesis suggests that sociability is the underlying learning factor of different grouping tactics. However, more work is needed to be able to formulate reliable explanations and recommendations. Experimental research is necessary to determine with certainty the causes of those observed effects. We believe that the positive effect of partnership emerges from social commitment. The socio-cognitive derivatives of loyalty such as trust, constancy, and fluid communication outweighs the costs of coordination and the reduction in the range of relationships that can be established.

The grouping strategies identified cannot be classified either into those social learning strategies that specify under which circumstances copy others, nor those that describe from whom individuals learn. However, by definition, they are social learning strategies due to the evidence of long-term changes in behavior caused by stimuli derived from observation and interaction with other individuals.

## Materials and methods

All games were downloaded from Conquer Club, a free service that offers to play RISK like games. The website allows any person, and not just registered participants, to explore the matches and browse their related data. Registered users are identified by their nicknames and, to be accepted as users, they have to agree with having their games stored in a publicly accessible server. Moreover, during the downloading process, each player is identified by an internal id number, anonymizing the data. In consequence, there is no need for individual consent due to this double layer of anonymity and the open nature of the website. We contacted Conquer Club’ owners to get authorization for performing this process, thus complying with this site’ terms of use.

The application consists of a Python script that connects to the Conquer Club server. The data between 2006/01/03 and 2009/07/12 is stored in a PostgreSQL database. There are near 4.4 million games played by almost 270 thousand different users.

To compute the skill, we use the TrueSkill 0.4.4 package for Python. All players start with a skill mean *μ* = 25 and a skill standard deviation of *σ* = 8.33. The draw probability value was set to 0 since there is no chance of drawing in Conquer Club.

### The game

#### Gameplay

**Fig 5:**
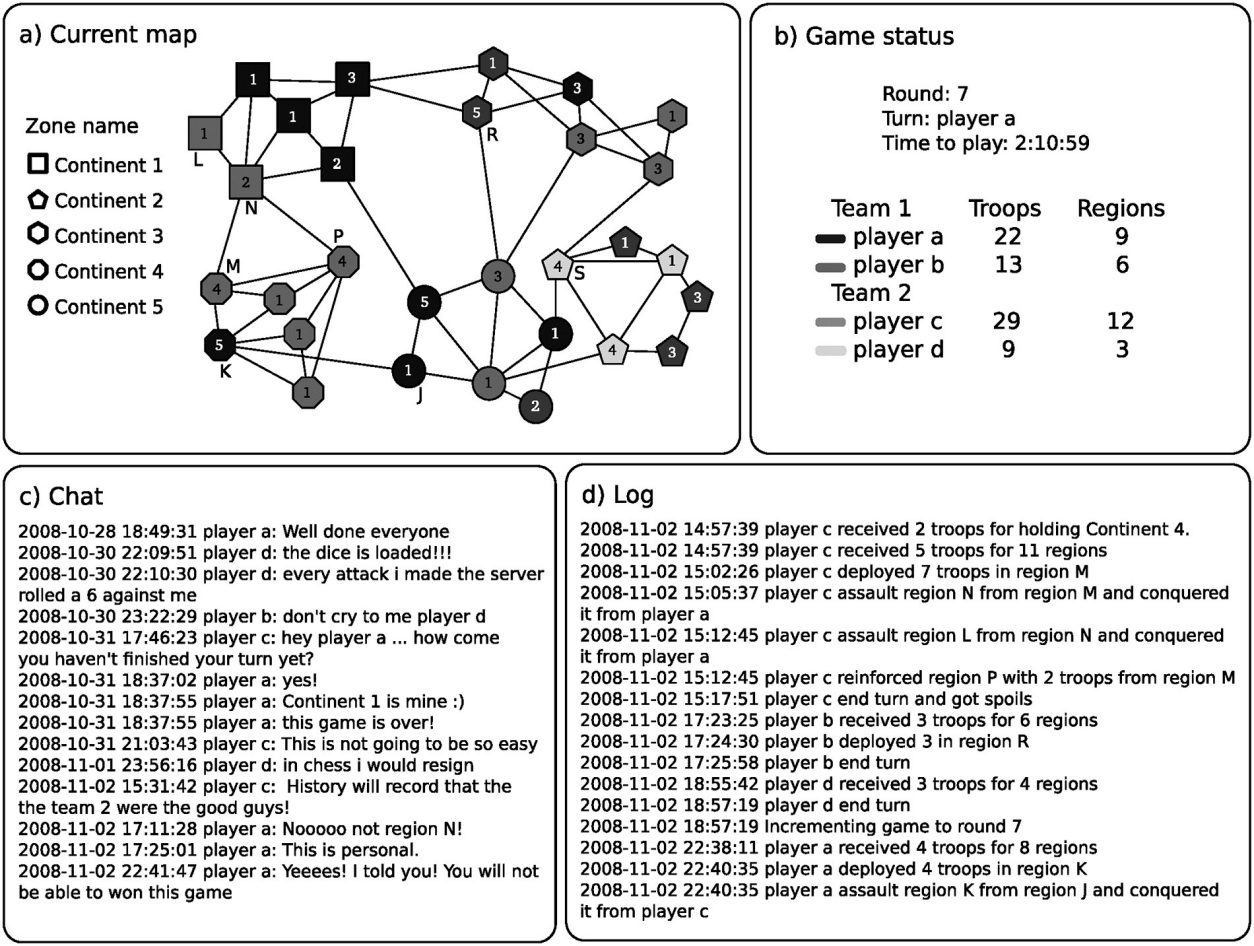
Scheme of Conquer Club game. a) The current game board showed as a graph with continents (regions of the same shape), players (regions of the same grayscale), and a number of troops in each region. The capital characters represent the names of the closest region. b) General game status: current round, the active player, and remaining time to play; and a summary of total troops and controlled regions for each player. c) Example of chat session during a game. d) Log of game used to extract game information with a scrapper.

At the beginning of a game, the regions of the selected map are randomly distributed among the players and populated with troops. Each turn consists of i) deploy new troops, ii) assault neighboring opponent’s regions, and iii) reinforce the regions. The game environment has four relevant elements: the current board, a panel with the game status, a public chat and a log of movements (Fig 5).

In this example, a double team game is played between two teams. The nodes of the graph represent the regions, the color indicates the player’s owner, the numbers represent the number of troops, and the shapes indicate to which zone it belongs. Alongside there is a panel with the round number, the active player, and the time remaining time to play the current round, and a summary of total troops and total controlled regions for each player. The color beside the nickname identifies each players’ regions in the map. A public chat service is available within each game. The Log records all movements of players, especially useful when players interact with the game sporadically.

At the beginning of every turn, the players earn new troops. The troop amount results from the number of occupied *regions* and the bonus of the controlled *zone* and eventually by the exchange of *spoils* (Section A in S1 File). We can see in the first line of the log in Fig 5d that player *c* received troops for holding Continent 4. These troops may be deployed at her occupied regions. For example, player *c* deploy all their troops at region *M*.

Once the deploy is finished the assault stage begins. A player can assault any opponent’s region as long as both are adjacent and the assaulting region has a minimum of two troops. The game engine rolls a die for each assaulting troop, except for one troop that needs to stay at the region, up to a maximum of three troops. Then, the system rolls a die for each defending troop, up to a maximum of two troops. The obtained values of each side are ordered increasingly and then compared one by one. If the assaulting dice is higher, then the defending region loses a troop. If the defending dice is higher or equal, then the assaulting region loses a troop. If the attacker destroys all the defending troops, some of the remaining troops have to be moved to occupy the newly conquered region. In our example, player *c* assaults region *N* from region *M* and conquers it from player *a*. Then, this player uses the recently moved troops to conquer another region.

When the player finishes the assaults, some troops can be used to reinforce the defending position. The player may move some (but not all) the troops from one of the owned regions to any other occupied and connected region. The reinforcements game configuration option determines how many of these reinforcement plays are allowed. In our example, player *c* reinforces region *P* by moving two troops from region *M*. Finally, player *c* finishes the turn and player *b* starts with her round.

#### Matchmaking

The platform has a “Join a Game” section, where all the players can see all the open games. When a player creates a game, she chooses: a) gameplay options, b) game type, free-for-all or team game, c) the number of participants, and d) the join method, public, public with reserved slots, and private. Public games are those to which anyone can join. The public games with reserved slots have slots assigned to particular players, and the rest are open to general players. Private games can be accessed by any player who has access to the game’s password.

In this platform, there is no skill matching mechanism based on the probability of winning of the players. There are an internal ranking and a point system that players can use as a reference to estimate the skill of others. The point system is updated as Δ=min(Loser'sscoreWinner'sscore20,100). However, they are not precise indicators of players’ skill and the probability of winning between opponents. The internal ranking is the conjunction between the number of games played and the points reached.

When a player selects a game, she can see the names of those who are already joined. An icon and a star appear next to the names. The icons represent the players’ ranking. The stars summarize the opinion about the player that some of her previous opponents reported. At the end of a game, players can report, on a scale of 1 to 5, the behavior of the rest of the players regarding Fair Play, Gameplay, and Attitude.

### Skill estimator

TrueSkill was inspired by Elo method, developed by Arpad Elo in 1959 and adopted in 1970 by the World Chess Federation (FIDE).

#### Elo

The main idea of the Elo system is to model the probability of an outcome game based on players’ skill *s*_*i*_, *s*_*j*_. The model assumes that, in each game, the players exhibit a performance, a hidden random variable normally distributed, *p*_*i*_ ∼ *N*(*s*_*i*_, *β*^2^), centered at their unknown true skill value with some constant noise. It is assumed that the player who exhibits the greater performance is the winner. Under these assumptions, we can infer in each game who had the highest performance by observing the game outcome (win/lose). Then, the probability that player *i* wins is *P*(*p*_*i*_ > *p*_*j*_ | *s*_*i*_, *s*_*j*_) = *P*(*p*_*i*_ − *p*_*j*_ > 0 | *s*_*i*_, *s*_*j*_).

**Fig 6:**
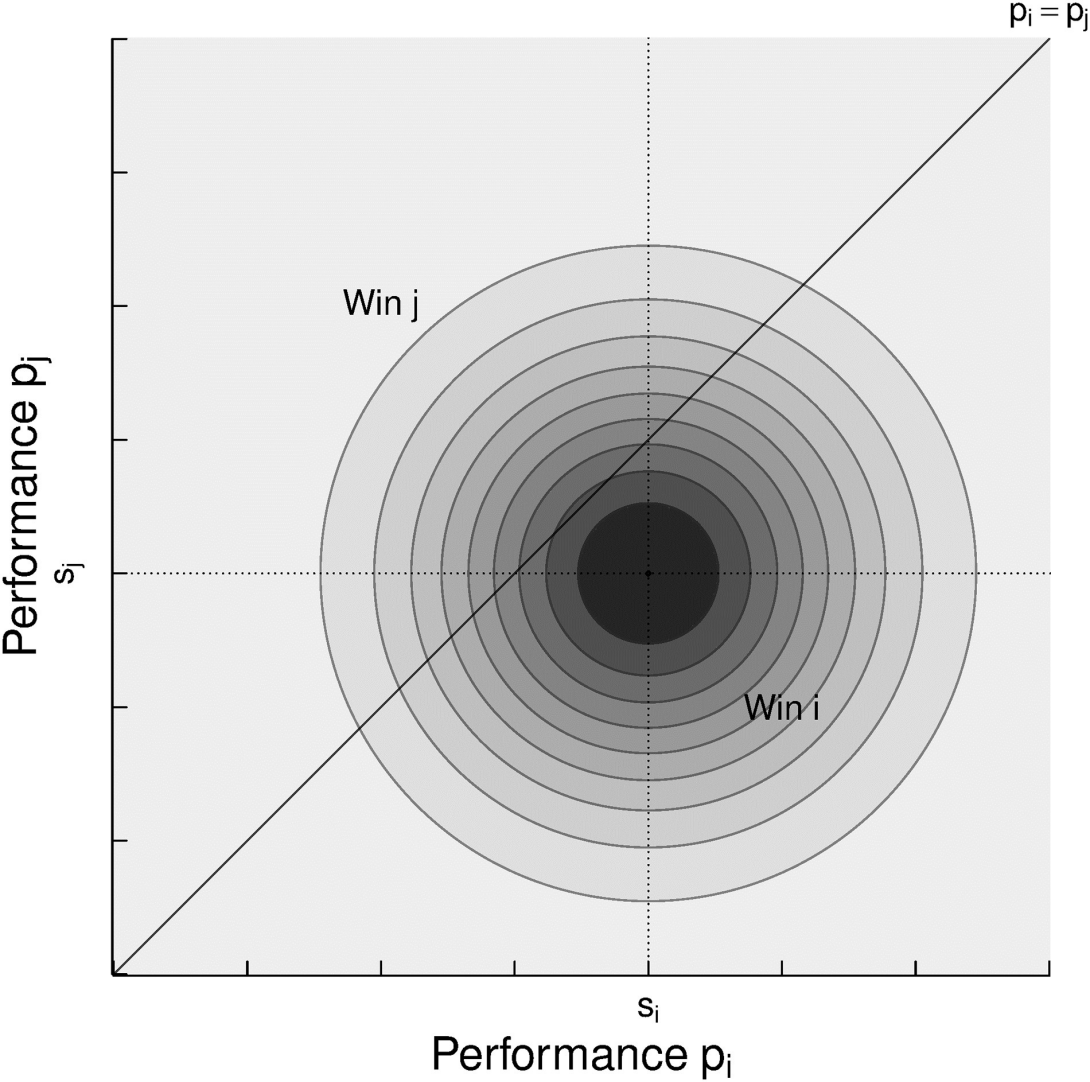
Joint probability of the performance of two players *i*, *j* under the assumption of *s*_*i*_ > *s*_*j*_ and independence. All lines parallel to the diagonal *p*_*i*_ = *p*_*j*_ represent “difference of performances” isobars *d*_*ij*_ = *p*_*i*_ − *p*_*j*_.

The “difference of performances” isobars *d*_*ij*_ = *p*_*i*_ − *p*_*j*_ are all lines parallel to the diagonal *p*_*i*_ = *p*_*j*_ at Fig 6. Then, the probability of a certain difference of performances *d*_*ij*_ is computed as,

$$P\left(d_{ij}|s_{i},s_{j}\right)=\;\int \int I\left(d_{ij}=p_{i}-p_{j}\right)N\left(p_{i};s_{i},\beta^{2}\right)N\left(p_{j};s_{j},\beta^{2}\right)\;dp_{i}\;dp_{j}$$ (6)

**Fig 7:**
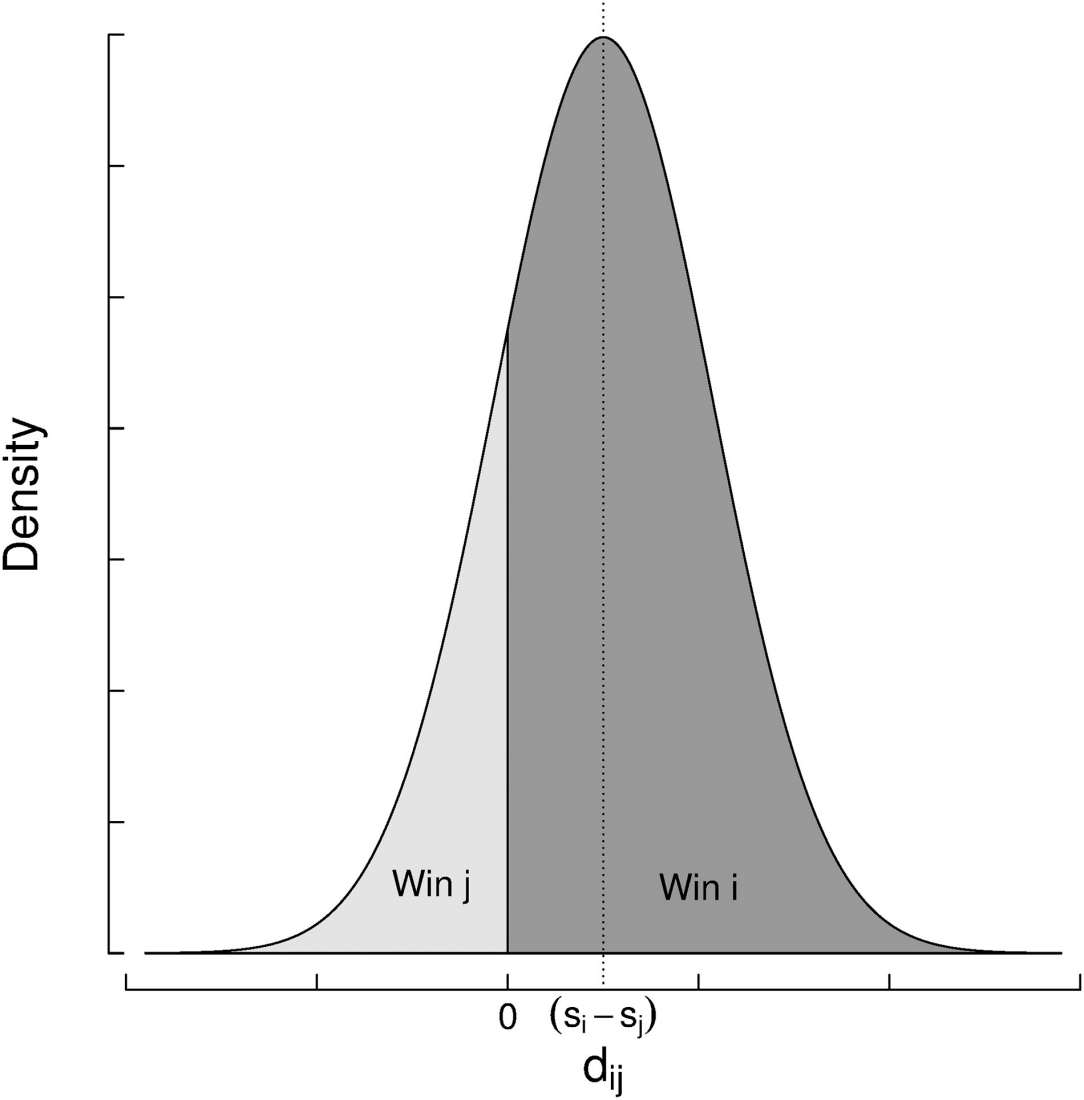
The probability of the outcome of a game under the assumptions of the Elo rating system with *s*_*i*_ > *s*_*j*_. The area under the curve in the positive interval (*d*_*ij*_ > 0) is the winning probability for the player *i*, and the area under the curve in the negative interval is the winning probability of player *j*.

It can be shown, based on Gaussians’ properties, that the difference of performance *d*_*ij*_ is also normally distributed, centered at the skill difference point with double variance (Fig 7).

$$P\left(d_{ij}|s_{i},s_{j}\right)=N\left(d_{ij};s_{i}-s_{j},2\beta^{2}\right)$$ (7)

This reduces the problem of computing the probability of the game outcome to a single-dimension problem related to the performance difference.

Let the result of a game $r_{ij}=I\left(d_{ij}>0\right)$. The probability of winning, *r*_*ij*_ = 1, can be computed as:

$$P\left(r_{ij}=1|s_{i},s_{j}\right)=P\left(d_{ij}>0|s_{i},s_{j}\right)=1-\Phi (\frac{0-(s_{i}-s_{j})}{\sqrt{2}\beta })⩮\Phi (\frac{s_{i}-s_{j}}{\sqrt{2}\beta })$$ (8)

where Φ is the cumulative distribution function of the standard normal distribution, *N*(0, 1). The highlighted equality (⩮) is derived by symmetry of the Normal density function. Then, the probability of the result can be written as:

$$P\left(r_{ij}|s_{i},s_{j}\right)=\left(r_{ij}\right)P\left(r_{ij}=1|s_{i},s_{j}\right)+\left(1-r_{ij}\right)P\left(r_{ij}=0|s_{i},s_{j}\right)$$ (9)

Now we can calculate the probability of the result given the skill estimates (*s*_*i*_, *s*_*j*_). Then, we have a reference to update them. Observing very unlikely results would indicate that the skills estimated so far are not entirely correct and should be updated to a greater extent than if the observed results were as expected.

$$\Delta =\underset{\begin{array}{c} \text{Direction}\\ \text{(Outcome)} \end{array}}{\underset{︸}{y_{ij}}}\underset{\begin{array}{c} \text{Magnitud}\\ \left(\text{Outcome}\;\text{Surprise}\right) \end{array}}{\underset{︸}{\left(1-P(r_{ij}|s_{i},s_{j})\right)}}$$ (10)

where the direction *y*_*ij*_ = 2*r*_*ij*_ − 1.

Finally the update in the Elo system is given by

$$s_{i}^{\text{new}}=s_{i}^{\text{old}}+K\Delta$$ (11)

where *K*, an arbitrary parameter, is the maximum number of points that are disputed in each game.

Even, there is a criterion to define it, this is one disadvantage of the Elo rating system. A second problem is that the estimated skill must be considered provisional until the player reaches a number, also arbitrary, of games. A third problem is that with Elo we cannot estimate the skill of players when playing in teams. The Bayesian model TrueSkill solves all these problems.

#### TrueSkill

The TrueSkill method [48] was introduced in 2006 by Ralf Herbrich and has two patents [52, 53]. TrueSkill shares the dependency model of the Elo rating system between skill, performance, and probability of winning. It extends it through a Bayesian model that incorporates a belief distribution of skills (prior), a model of team performance and a non-arbitrary update function (the posterior distribution).

#### Skill

One of the novelties of TrueSkill is the notion of the uncertainty of the skill estimation. The skill estimate *s*_*i*_, previously represented by a scalar, is now represented as a prior distribution of beliefs with normal density function.

$$s_{i}∼N\left(\mu_{i},\sigma_{i}^{2}\right)$$ (12)

where *μ* and *σ* initially acquire arbitrary values.

This is not an issue. What matters about the average is not its absolute value but the difference with other players. On the other hand, the standard deviation should be large enough to represent the uncertainty that we actually have with respect to the average. In general, *μ* = 25 and $\sigma =\frac{25}{3}$ are used as initial values.

#### Performance

As in the Elo system, it is assumed that the final outcome of the game depends on the *p*_*i*_ performance of the players,

$$p_{i}∼N\left(s_{i},\beta^{2}\right)$$ (13)

Now the probability of a given performance *p*_*i*_ is defined as,

$$P\left(p_{i}|\mu_{i},\sigma_{i}\right)=\int N\left(p_{i};s_{i},\beta^{2}\right)N\left(s_{i};\mu_{i},\sigma_{i}^{2}\right)ds_{i}$$ (14)

**Fig 8:**
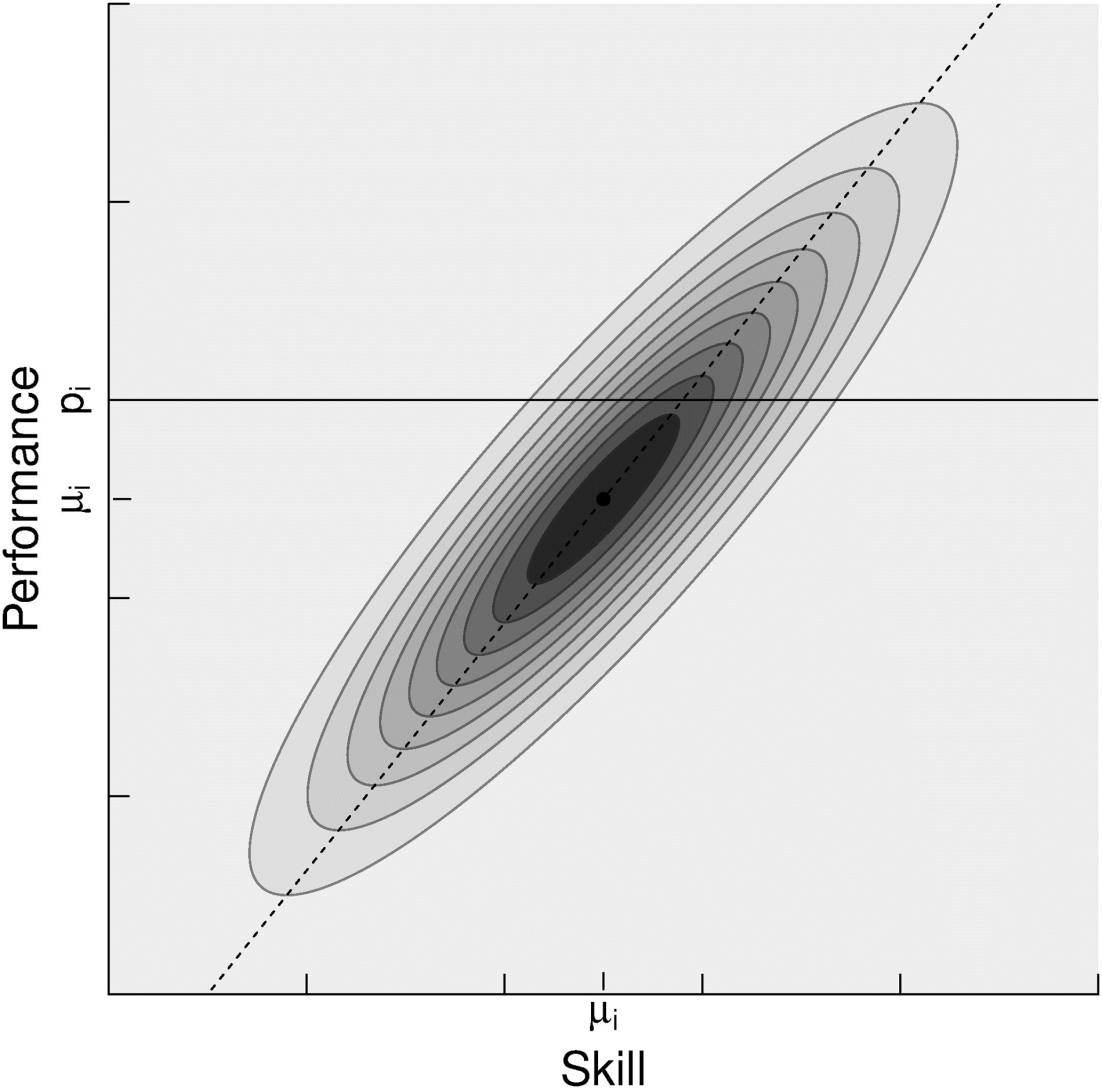
Performance distributions, *N*(*p*_*i*_; *s*_*i*_, *β*^2^) weighted by the probability of the skill belief distribution $N(s_{i};\;\mu_{i},\sigma_{i}^{2})$. The area under the solid line must be integrated to compute a certain probability *p*_*i*_.

Then, we can compute the probability of a given performance, *p*_*i*_, by integrating the area under the solid line in Fig 8. We rewrite the integral 14 using the symmetry property, *N*(*x*; *μ*, *σ*^2^) = *N*(*μ*; *x*, *σ*^2^).

$$P\left(p_{i}|\mu_{i},\sigma_{i}\right)=\int N\left(s_{i};p_{i},\beta^{2}\right)N\left(s_{i};\mu_{i},\sigma_{i}^{2}\right)ds_{i}$$ (15)

It can be shown (S2 File) that the product of Gaussians is also normally distributed,

$$P\left(p_{i}|\mu_{i},\sigma_{i}\right)=\int \underset{\text{Scalar}\;\text{independent}\;\text{of}\;s_{i}}{\underset{︸}{N(p_{i};\mu_{i},\beta^{2}+\sigma_{i}^{2})}}N\left(s_{i},\mu_{*},\sigma_{*}^{2}\right)\;ds_{i}=N\left(p_{i};\mu_{i},\beta^{2}+\sigma_{i}^{2}\right)$$ (16)

#### Teams

The second novelty of TrueSkill enables to update the players’ skill when they play team games. TrueSkill model states that one team beats another when their team’s performance is greater than the opponent team’s performance. With *A* the partition of players (the team assignment), the performance of a team is defined as the sum of the performances of its members,

$$t_{e}=\sum_{j\in A_{e}}p_{j}$$ (17)

Then, the probability of a given team’s performance is defined as

$$P\left(t_{e}|A_{e}\right)=\int \cdots \int I\left(t_{e}=\sum_{j\in A_{e}}p_{j}\right)(\prod_{i\in A_{e}}N\left(p_{i};\mu_{i},\beta^{2}+\sigma^{2}\right))d\vec{p}$$ (18)

The team’s performance assumption is only used to adopt the skill of individual players such that the team outcome can be best predicted based on the additive assumptions of the skills. The empirical probability distribution of individual and team games are exactly the same based on a Kolmogorov-Smirnov test, showing that the skill estimated by simple addition preserves the probability of winning based on this measure of team skill (Fig A in S1 File).

**Fig 9:**
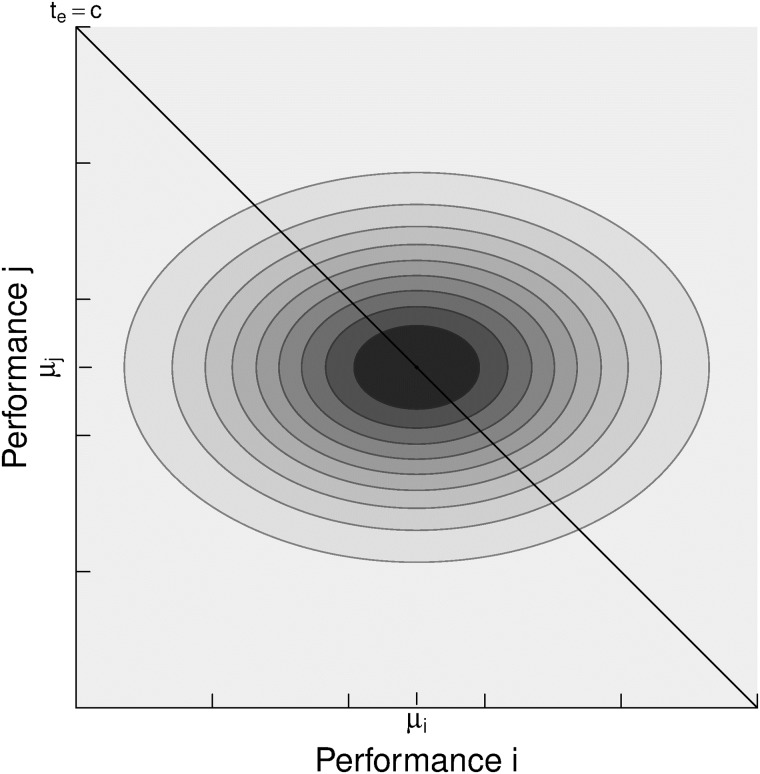
Joint probability of the performance of two teammates *i*, *j*. Lines parallel to the diagonal *t*_*e*_ = *c* represent team performance isobars.

Mathematically, a team’s performance with two players we can see graphically in Fig 9. To compute the probability of a given team’s performance *c* we must integrate the area under the corresponding isobar, *t*_*e*_ = *c* (See Fig 9).

It can be shown in a team with 2 members (S2 File),

$$\begin{array}{c} \begin{array}{cc} P(t_{e}|A_{e}=\left\{i,j\right\}) & =\int N\left(p_{i}\;;\;\mu_{*},\sigma_{*}^{2}\right)\overset{\text{Scalar}\;\text{independent}\;\text{of}\;p_{i}}{\overset{︷}{N(t_{e}\;;\;\mu_{i}+\mu_{j},2*\beta^{2}+\sigma_{i}^{2}+\sigma_{j}^{2})}}dp_{i}\\ & =N(t_{e}\;;\;\mu_{i}+\mu_{j},2\beta^{2}+\sigma_{i}^{2}+\sigma_{j}^{2}) \end{array} \end{array}$$ (19)

It can be shown by induction (S2 File) that in a team with *n* members the probability of a given team’s performance is:

$$P\left(t_{e}|A_{e}\right)=N(t\;;\;\sum_{i\in A_{e}}\mu_{i},\sum_{i\in A_{e}}\beta^{2}+\sigma_{i}^{2})$$ (20)

#### Difference

The difference teams’ performances are what determines the outcome of the game.

$$d_{ab}=t_{a}-t_{b}$$ (21)

In the same way, as in the Fig 6, the difference of performance isobars are the lines parallel to the diagonal of zero difference. To compute the probability of a given difference of performance *d*_*ab*_ is:

$$\begin{array}{cc} P(d_{ab}|A_{a},A_{b})= & \int \int I\left(d_{ab}=t_{a}-t_{b}\right)\cdot N\left(t_{a};\sum_{i\in A_{a}}\mu_{i},\sum_{i\in A_{a}}\beta^{2}+\sigma_{i}^{2}\right)\cdot \\ & \;N\left(t_{b};\sum_{i\in A_{b}}\mu_{i},\sum_{i\in A_{b}}\beta^{2}+\sigma_{i}^{2}\right)\;dt_{a}dt_{b} \end{array}$$ (22)

It can be shown (S2 File) that them probability of a given difference of performance *d*_*ab*_ is:

$$\begin{array}{c} P\left(d_{ab}|A_{a},A_{b}\right)=N(d_{ab}\;;\;\underset{\text{Expected}\;\text{difference}\;(\delta )}{\underset{︸}{\sum_{i\in A_{a}}\mu_{i}-\sum_{i\in A_{b}}\mu_{i}}},\underset{\text{Total}\;\text{variance}\;(\vartheta )}{\underset{︸}{\sum_{i\in A_{a}\cup A_{b}}\beta^{2}+\sigma_{i}^{2}}})=N\left(d_{ab}\;;\;\delta ,\vartheta \right) \end{array}$$ (23)

#### Result

A win of a team *a* over another team *b* is modeled as:

$$r_{ab}=d_{ab}>0$$ (24)

Then, the probability of victory of a team over another is computed as:

$$P\left(r_{ab}=\text{True}|A_{a},A_{b}\right)=P\left(d_{ab}>0|A_{a},A_{b}\right)=\Phi (\frac{\delta }{\sqrt{2\vartheta }})$$ (25)

The observed outcome of a game it is modeled with an ordered vector of teams, *o*, such that $t_{o_{1}}<\cdots <t_{o_{\left|A\right|}}$.

#### Posterior

**Fig 10:**
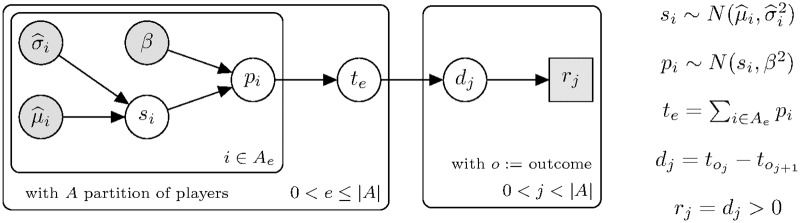
Bayes network of TrueSkill method.

In summary, the TrueSkill model can be represented by a graphical network (Fig 10).

From Bayes rule, we obtain the posterior distribution,

$$P\left(s|o,A\right)=\frac{P(o|s,A)P(s)}{P(o|A)}$$ (26)

**Fig 11:**
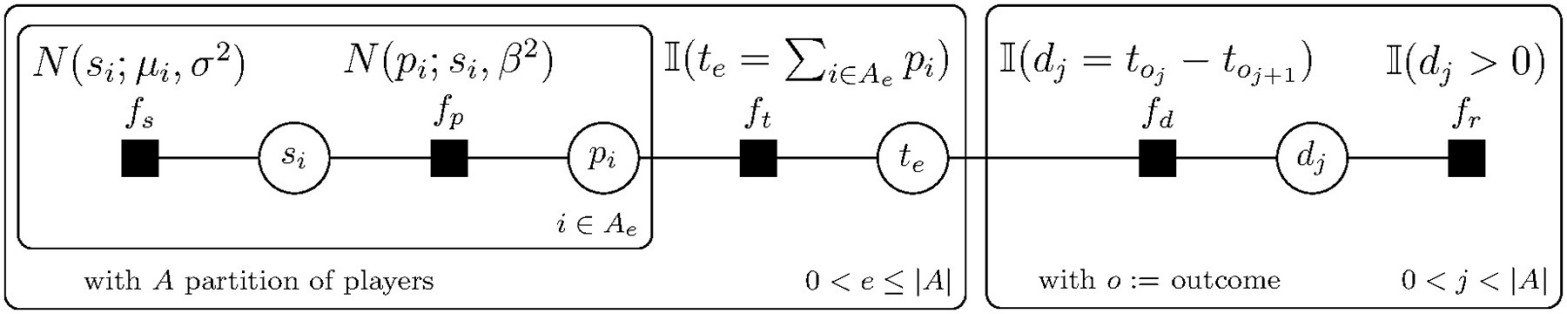
Bayes factor network of TrueSkill method.

The exact posterior could be computed by performing the sum-product algorithm [49] over the factor graph (Fig 11).

**Fig 12:**
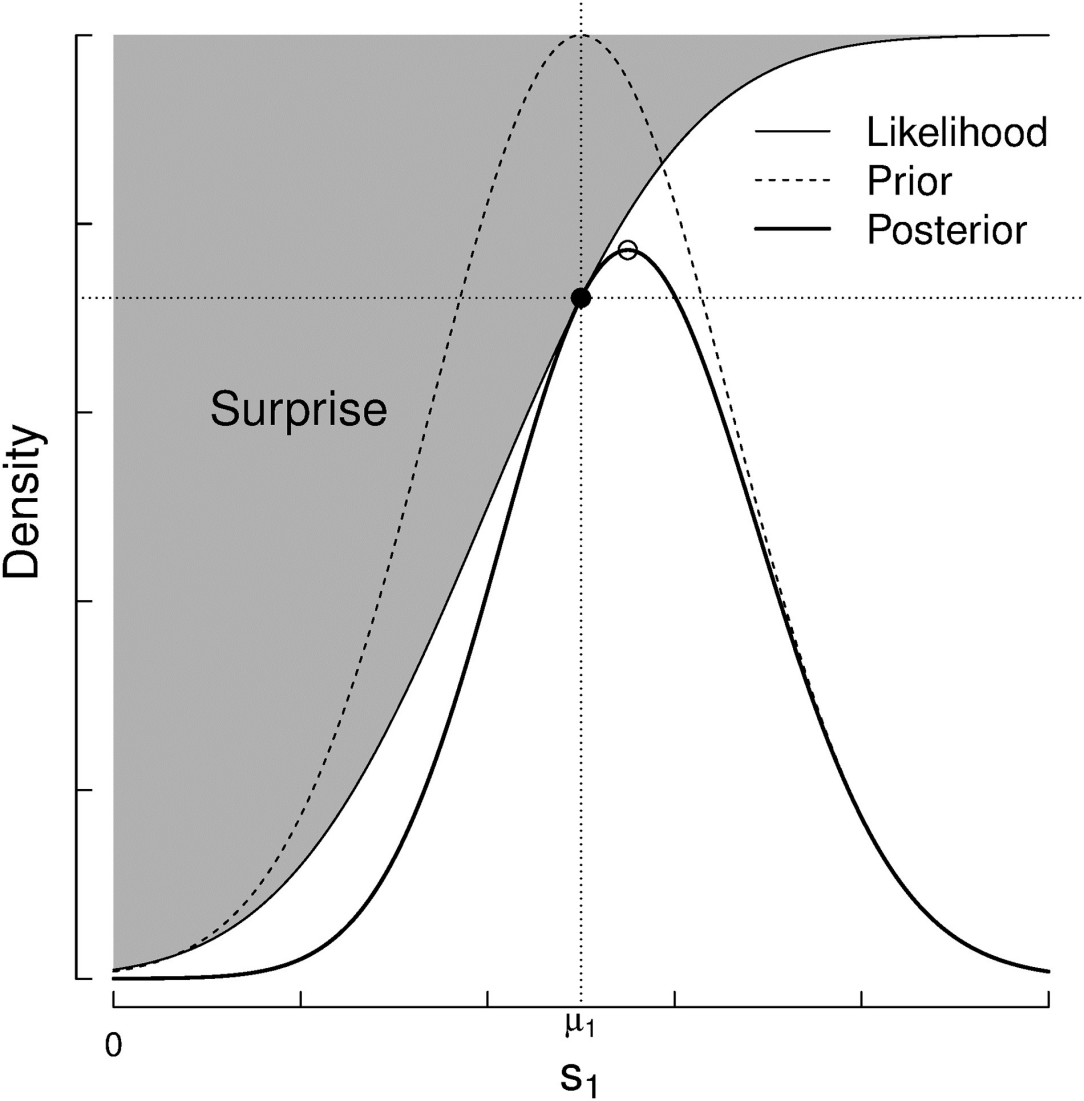
TrueSkill update procedure for the winning case, where *δ* is the expected difference between teams.

**Fig 13:**
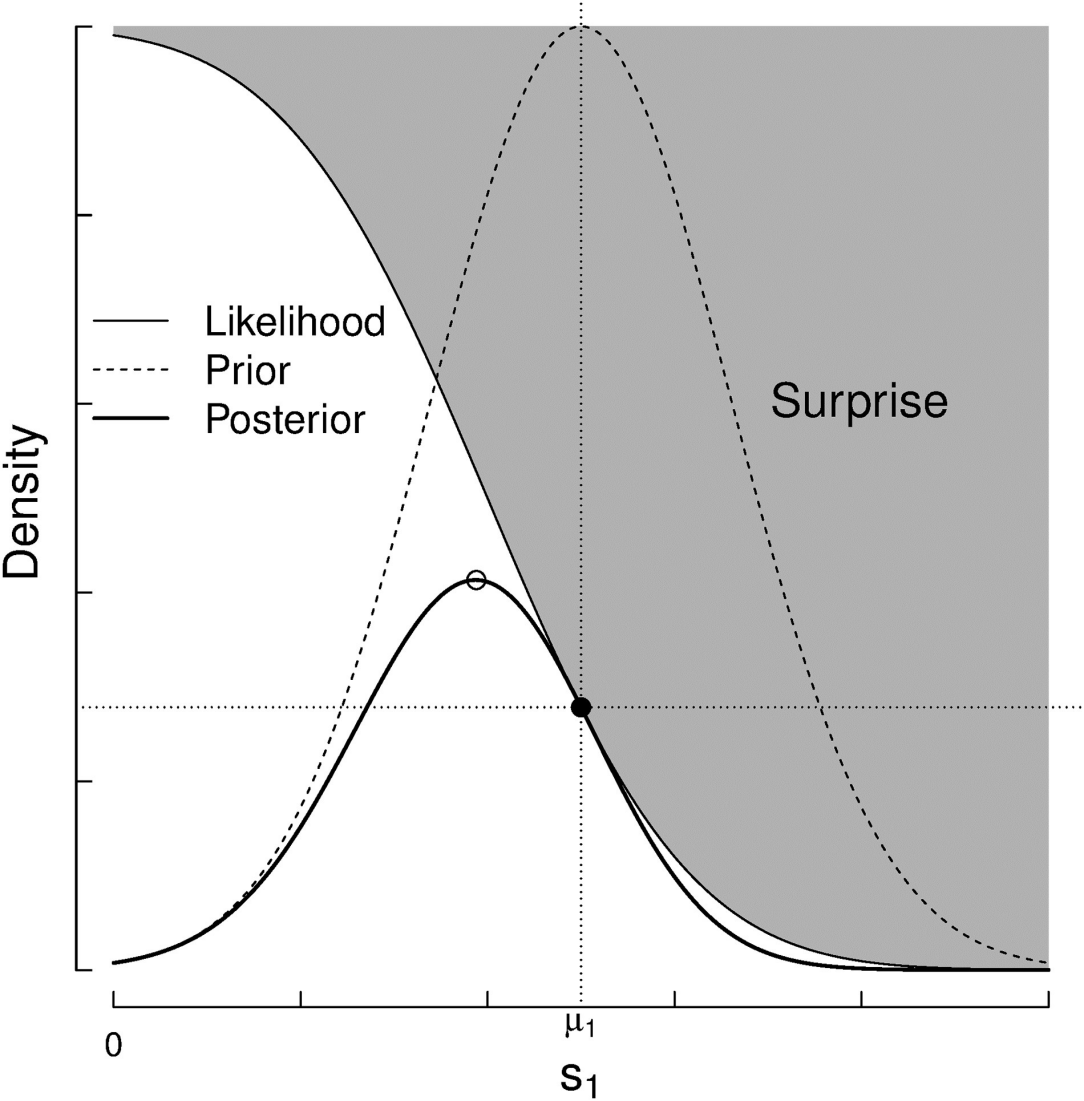
TrueSkill update procedure for losing case, where *δ* is the expected difference between teams.

With two teams, the exact posterior update function is (See all the details S2 File),

$$\begin{array}{c} P\left(s_{i}|o,A\right)\propto \{\begin{array}{cc} N\left(s_{i}\;;\;\mu_{i},\sigma_{i}^{2}\right)\;\Phi \left(0\;;\;\delta \left(s_{i}\right),\vartheta -\sigma_{i}^{2}\right) & \text{Winning}\;\text{case}\\ N\left(s_{i}\;;\;\mu_{i},\sigma_{i}^{2}\right)\;\left(1-\Phi \left(0\;;\;\delta \left(s_{i}\right),\vartheta -\sigma_{i}^{2}\right)\right) & \text{Losing}\;\text{case} \end{array} \end{array}$$ (27)

where *δ*(*s*_*i*_) = *δ* − *μ*_*i*_ + *s*_*i*_, the expected difference between teams replacing the estimated skill (*μ*) by their true skill *s*_*i*_ (Figs 12 and 13).

Finally, TrueSkill takes as the posterior of the model the Gaussian that minimizes the KL divergence with the exact posterior.

## Statistical information

We performed a Wilcoxon rank-sum test at Figs 1, 2, 3, and at Tables A and B in S1 File. A Wilcoxon confident interval was performed at Figs 2 and 3. We performed a multiple linear regression at Table 1, and at Fig F in S1 File. We performed a general linear mixed model at Table 2. We performed a two sided Kolmogorov-Smirnov tests at Figs B and G in S1 File. Tests were performed using R version 3.0.2 stats package, and Python version 3.6 statsmodels package.

## Supporting information

S1 File Details about the gameplay: Rules, maps, limits and mechanics. Fig A: The probability of winning as a function of the skill difference between: (a) Case of two individual opponents and two team opponents. (c) Case of three opponents. Fig B: Histogram of skill. Fig C: Learning curve of committed population. Fig D: Learning curve of population of players without team games played. Fig E: Learning curve of loyal and casual subclasses for medium (a) and weak (b) team-oriented behavior. Fig F: Influence of loyalty, TOB, and the faithfulness interaction over skill acquisition. Fig G: Team probability of winning at function of skill difference between teams and between teammates. Table A: Significance difference between distributions of skill after the first game played at Fig 1. Table B: Significance difference between strong, medium and weak team-oriented population.
(PDF)

S2 File TrueSkill, Technical Report. Analytical computation of the posterior distribution. This file includes details about update rules and the derivation of used expressions.
(PDF)
