# Supplementary material for: Faithfulness-boost effect: Loyal teammate selection correlates with skill acquisition improvement in online games
Source: PLoS One. 2019 Mar 5;14(3):e0211014. doi: 10.1371/journal.pone.0211014 (PMC6400383; doi:10.1371/journal.pone.0211014)
Supplement: S1 File — Fig A: The probability of winning as a function of the skill difference between: (a) Case of two individual opponents and two team opponents. (c) Case of three opponents. Fig B: Histogram of skill. Fig C: Learning curve of committed population. Fig D: Learning curve of population of players without team games played. Fig E: Learning curve of loyal and casual subclasses for medium (a) and weak (b) team-oriented behavior. Fig F: Influence of loyalty, TOB, and the faithfulness interaction over skill acquisition. Fig G: Team probability of winning at function of skill difference between teams and between teammates. Table A: Significance difference between distributions of skill after the first game played at Fig 1. Table B: Significance difference between strong, medium and weak team-oriented population. (PDF) [file pone.0211014.s001.pdf]

Supplementary Information S1 of “Faithfulness-boost effect: loyal teammate selection correlates with skill acquisition improvement in online games”

Gustavo Landfried[1,2] Diego Fernández Slezak[1,2,*] Esteban Mocskos[1,3,*]

1 Universidad de Buenos Aires. Facultad de Ciencias Exactas y Naturales. Departamento de Computación. Buenos Aires, Argentina
2 CONICET-Universidad de Buenos Aires. Instituto de Investigación en Ciencias de la Computación (ICC). Buenos Aires, Argentina
3 CONICET. Centro de Simulación Computacional p/Aplic Tecnológicas (CSC). Buenos Aires, Argentina

* dfslezak@dc.uba.ar, emocskos@dc.uba.ar

A Gameplay options

When a member of Conquer Club chooses to start a new game, she has to select between different options which affect the game development. Next, we introduce and describe briefly these options.

Number of Players A game can have between 2 and 12 players.

Map The map defines the amount of regions and zones, and the connection between them. Conquer Club offers more than 200 maps, some of them are tagged as *beta*, which could lead to an unbalance gameplay and could be modified anytime by the service administrators. When starting a game, the player has to select a map or let the system choose one randomly.

Game Types Each game can be single or team based. The single game type affects the way the game points are accounted and the target to be achieved. The tame based games specifies the amount of players by team from 2 up to 4 players each team. In team games, each player can deploy troops on teammate's regions and reinforce teammate's adjacent regions. The score is calculated per team.

Initial Troops The initial troop population can be select between automatic or manual. In the first one, each region is initially populated with 3 troops. With the second option, each region is initially populated with 1 troop and during the first round of the game each player has to deploy the rest. If the player misses the first round, the troops are automatically deployed by the system.

New Troops In each round, every player can reinforce the regions. The amount of available troops is determined by

$$\text{troops} = \min\left(\lfloor\frac{\text{regions}}{3}\rfloor, 3\right) + \text{zone bonus} + \text{spoils bonus} \quad (1)$$

where *regions* is the amount of regions controlled by the player, *zone bonus* is the bonus of troops due to the control of zone by a single player, and *spoils* are special bonuses which can be get when conquering regions: each time a player conquers a regions it get one spoil which can be of one of three predefined colors; when a player get three spoils of the same color, can be exchanged for additional troops.

Play Order In a sequential game player the registration order is followed. With teams based games the order is alternating between teams. In a freestyle players can play simultaneously. After the last player takes her turn, a new round begins. The last player (or team) to end their turn in each round is blocked from taking a second back-to-back turn and must wait until either an opponent begins their turn or half of the round passes.

Spoils Escalating type worth growth almost linear (by steps of 2, 3 and 4) until 100. Then it begins to escalate more rapidly. Flat Rate sets fixed worth. Nuclear and Zombie sets are not worth any troops.

Reinforcements At chained option, players can reinforce once and following connected and owned regions. At adjacent option, players can reinforce once and only from a direct neighbor. At Parachute option, players can reinforce once between any territory of their own. AT unlimited option, players can reinforce as many times as you like from regions connected by regions you own. With No reinforcements, players cannot reinforce at all.

Special Gameplay: Fog of War The fog of war option masks enemy positions that are not adjacent of players forces (or their team's forces).

Special Gameplay: Trench Warfare With trench warfare players can only assault from regions held continuously since the start of your turn. If a player conquer a region (or re-conquer a region) they cannot assault further from that region during the same turn. The one exception being 'killer neutrals' from which players may continue assaulting.

Round Limit With round limits, the game will automatically finish at the end of the specified round. The winner will be the surviving player with the most troops. If there is a tie, it will be broken based on the most regions. If there is still a tie, the winner is selected based on join order. When playing a round limited game with teams, the winning team will be based on combined team troop count.

Round Length Most games on Conquer Club are casual games with 24 hour rounds. There are also speed games with round lengths ranging from 5 minutes to 1 minute. These real-time games automatically refresh and an extra 2.5 minutes is added to the round length when someone is eliminated. If a player logouts or their session is idle for more than 15 minutes they will be automatically dropped from any waiting speed games.

B Supplementary figures

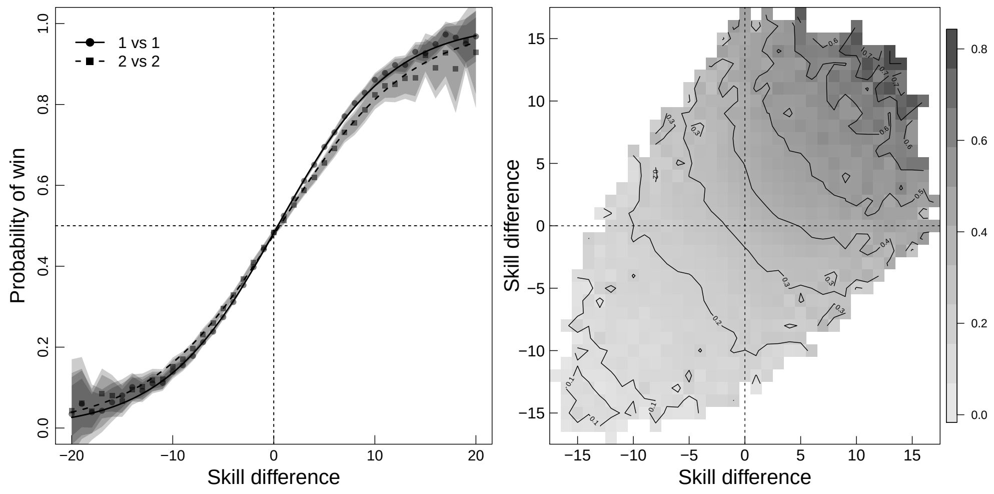

(a) **(b)**

Fig A. Probability of win at function of difference skill between. (a) Case of two individual opponents and two team opponents. Both figures shows exactly the same distribution based on a Kolmogorov–Smirnov test (two samples $D = 0.073$ *p-value*$= 0.9999$), showing that the skill estimated by simple addition preserves the probability of winning based on this measure of team skill. Fitted $\frac{e^{\beta_1+\beta_2 \cdot x}}{1+e^{\beta_1+\beta_2 \cdot x}}$ is shown. (b) Case of three opponents. Contour lines of probability of win are shown.

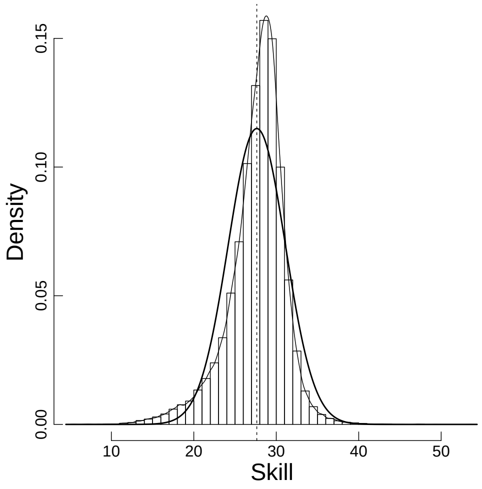

Fig B. Histogram of skill at final snapshot (12-07-2009). The empirical distribution of skill does not follow a normal distribution. A one sample Kolmogorov-Smirnov between histogram and the normal distributions with empirical mean and variance reject the null hypothesis that the sample is drawn from the reference distribution ($D = 0.0599$ and *p-value*≈ 0).

n	Interval	Size	9	8	7	(b)	6	(a)	5	4	3
10	1024-∞	2997	0	0	0	0	1	1	1	1	1
9	512-1024	3141		0	0	0	0	0	1	1	1
8	256-512	5410			0	0	1	1	1	1	1
7	128-256	8490				0	0	1	1	1	1
(b)	(90-128)	5624					0	0	1	1	1
6	64-128	12108						0	1	1	1
(a)	(64-90)	6484							0	1	1
5	32-64	16481								1	1
4	16-32	20746									1
3	8-16	25319									

Table A. Analysis of significance difference by a Wilcoxon rank-sum test between distributions of skill after the first game played at Fig. A. 0 represents a non-significant difference between pairs of subpopulations while 1 represents a significant difference. We split the subpopulation by the number of games played (1) players with at least 8 games and less than 16 (2) players with at least 16 games and less than 32, and so on. Each subpopulation has the first 2^n games played of the subpopulation with at least 2^n games played and less than 2^{n+1}. For a more detailed analysis, we provided two extra cases that arise from dividing the subpopulation with at least 64 games and less than 128 into (a) players with at least 64 games and less than 90, and (b) players with at least 90 games and less than 128.

TOB	size	p-value (vs Weak)	p-value (vs Medium)
Strong	103	$4.5e^{-17}$	$1.1e^{-5}$
Medium	271	$6.7e^{-9}$	
Weak	3845		

Table B. We perform a Wilcoxon rank-sum test between the skill empirical distribution after 500 games played of the strong, medium and weak team-oriented population.

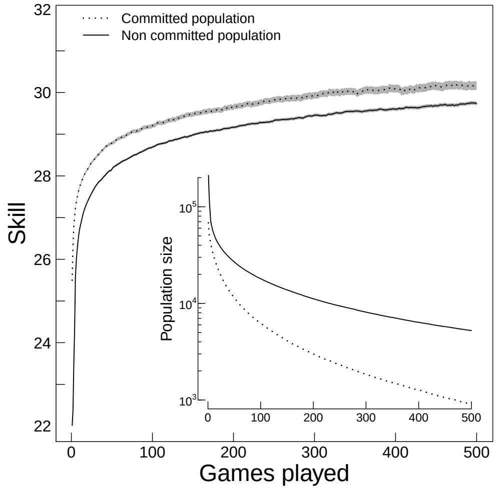

Fig C. Learning curve of the committed population (i.e. players with all games finished) and non-committed population (i.e. players with at least one game not finished). Skill average is reported. The band represents 2 times the standard error.

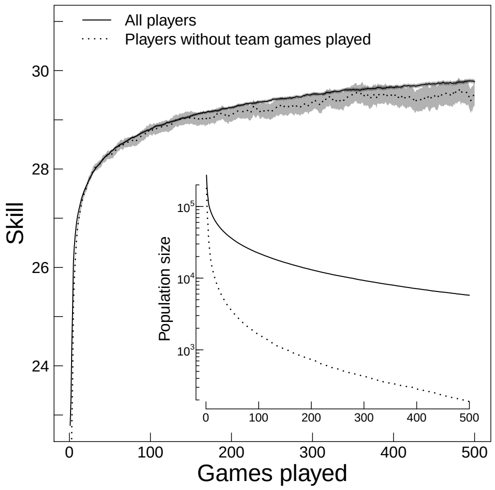

Fig D. Learning curve of the population of players without team games played. For reference, we show the learning curve of the whole population. Skill average is reported. The band represents 2 times standard error.

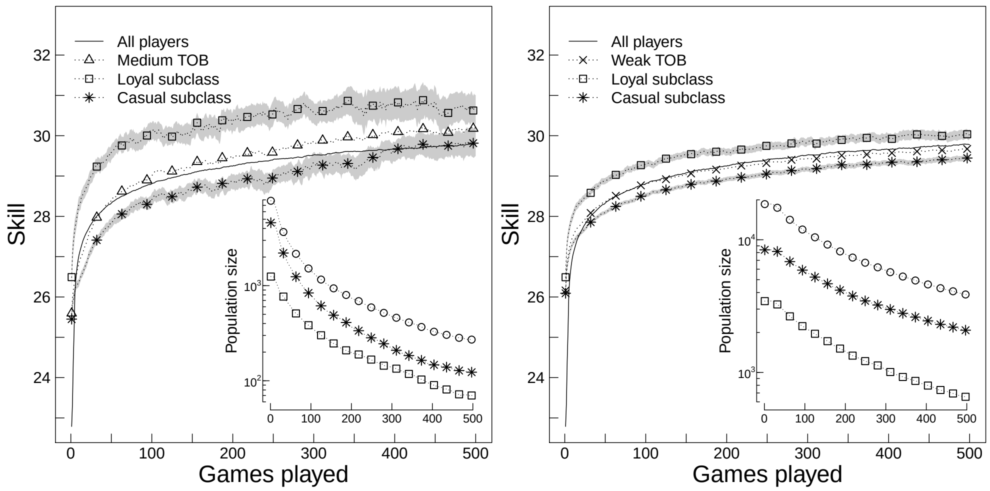

(a) Medium TOB **(b)** Weak TOB

Fig E. Learning curve of loyal and casual subclasses for medium (a) and weak (b) team-oriented behavior. For reference, we show the learning curve of the whole population and team-oriented behavior. The average skill is reported. The band represent 2 times the standard error.

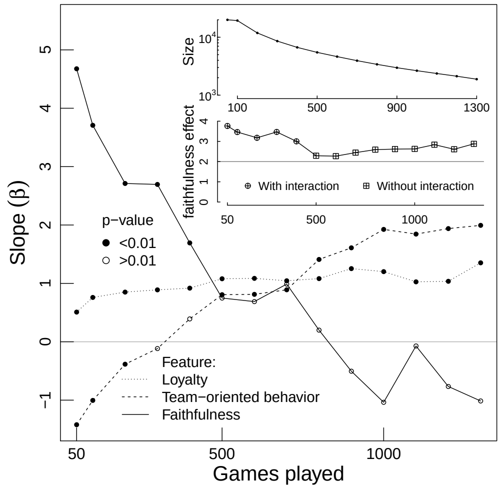

Fig F. Influence of loyalty, TOB, and the faithfulness interaction over skill acquisition for players with same experience. Model: $\mathrm{skill}_i \sim \beta_1 \mathrm{loyalty}_i + \beta_2 \mathrm{TOB}_i + \beta_3 \mathrm{faithfulness}_i$. We build a linear model every 100 games played, from 100 to 1300 games played. We add an extra population with 50 games played. The top subplot shows the population size at each point of experience. The lower subplot indicates the contribution to skill acquisition when faithfulness = 1 (i.e the faithfulness effect). We report the contribution of the model with interaction until 400 games played when faithfulness cease to be significant. After 400 games played we report the contribution of the model without interaction (i.e. $\mathrm{skill}_i \sim \beta_1 \mathrm{loyalty}_i + \beta_2 \mathrm{TOB}_i$).

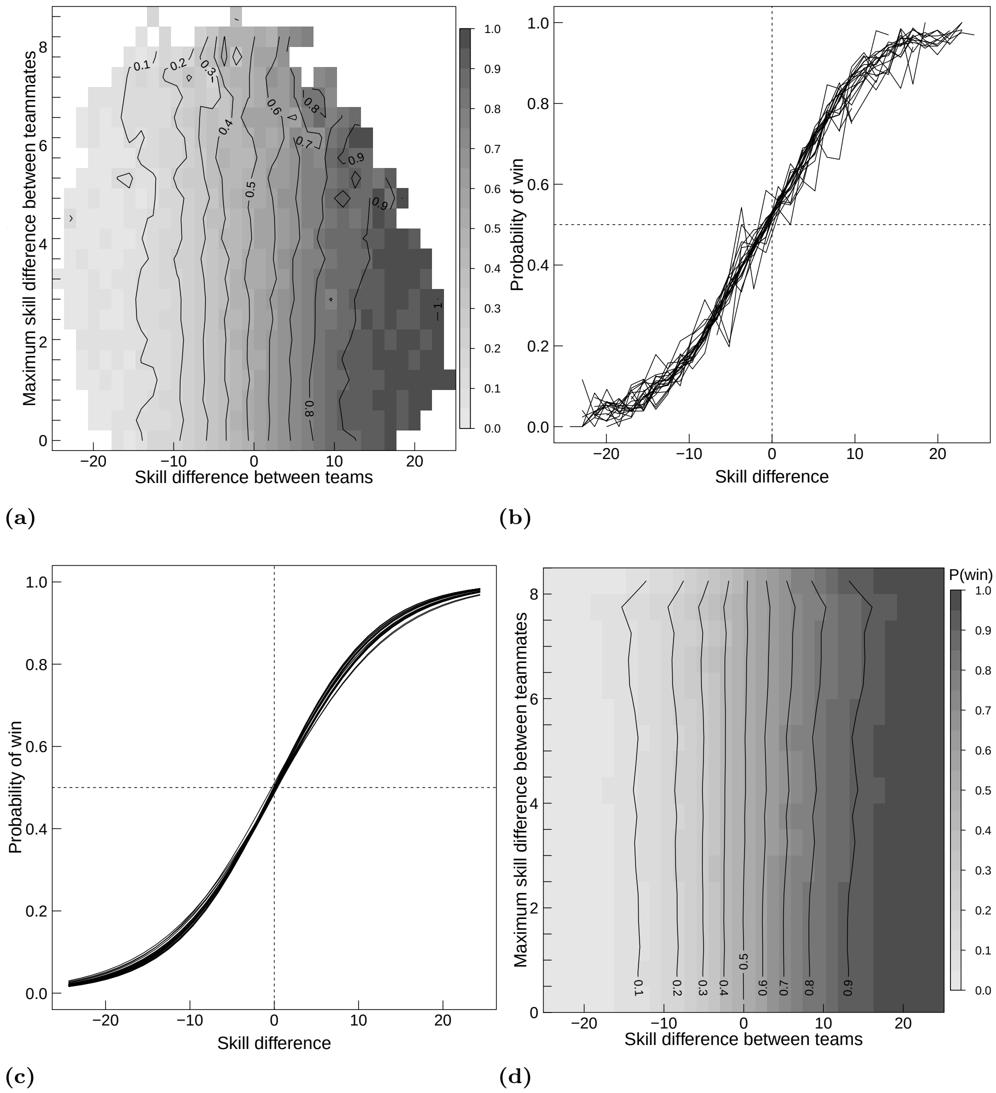

Fig G. Team probability of winning as a function of skill difference between teams and between teammates. (a) The team winning probability as a function of skill difference between teams and teammates. Each bin reports the empirical average probability of winning. Each non empty bin has at least 32 games played. (b) Each curve corresponds to a fixed difference between teammates showing the empirical team winning probability as a function of the difference between teams. The arbitrary bin disposition alters the shape of the curves. (c) Each curve corresponds to a fixed interval of difference between teammates showing the fitted team winning probability as a function of the difference between teams. (d) The fitted team winning probability as a function of the skill difference between the team and teammates. Each bin shows the value of the fitted curves at Fig. Gc.
