# Supplementary material for: Faithfulness-boost effect: Loyal teammate selection correlates with skill acquisition improvement in online games
Source: PLoS One. 2019 Mar 5;14(3):e0211014. doi: 10.1371/journal.pone.0211014 (PMC6400383; doi:10.1371/journal.pone.0211014)
Supplement: S2 File — Analytical computation of the posterior distribution. This file includes details about update rules and the derivation of used expressions. (PDF) [file pone.0211014.s002.pdf]

Supplementary Information S2 of "Faithfulness-boost effect: loyal teammate selection correlates with skill acquisition improvement in online games"

Gustavo Landfried[1,2] Diego Fernández Slezak[1,2,] Esteban Mocskos[1,3,*]

1 Universidad de Buenos Aires. Facultad de Ciencias Exactas y Naturales. Departamento de Computación. Buenos Aires, Argentina
2 CONICET-Universidad de Buenos Aires. Instituto de Investigación en Ciencias de la Computación (ICC). Buenos Aires, Argentina
3 CONICET. Centro de Simulación Computacional p/Aplic Tecnológicas (CSC). Buenos Aires, Argentina

* glandfried@dc.uba.ar

A TrueSkill: Technical Report

Analytical computation of the posterior distribution

To compute the posterior of the TrueSkill model [1], we use a generic message passing algorithm for factor graphs called sum-product algorithm [2].

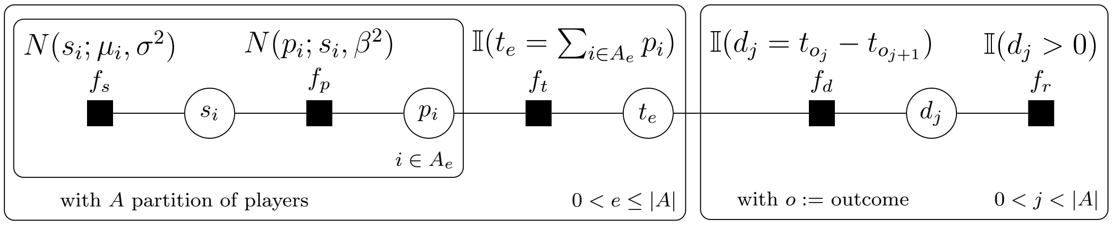

Fig A. Factor graph of TrueSkill

The Sum-Product Update Rule The message sent from a node v on an edge e is the product of the local function at v (or the unit function if v is a variable node) with all messages received at v on edges other than e, summarized for the variable associated with e.

Let $m_{x\to f}(x)$enote the message sent from node x to node f in the operation of the sum-product algorithm, and let $m_{f\to x}(x)$ denote the message sent from node f to node x. Also, let $n(v)$ denote the set of neighbors of a given node v. hen the message computations performed by the sum-product algorithm may be expressed as follows:

$$m_{x\to f}(x) = \prod_{h \in n(x) \setminus \{f\}} m_{h\to x}(x) \tag{1}$$

$$m_{f\to x}(x) = \int \cdots \int \Big(f(\boldsymbol{x}) \prod_{h \in n(f) \setminus \{x\}} m_{h\to f}(h) \Big) \, d\boldsymbol{x}_{\setminus x} \tag{2}$$

where $\boldsymbol{x} = \arg(f)$ is the set of arguments of the function f. Then, to calculate any marginal,

$$g_i(x_i) = \prod_{h \in n(x_i)} m_{h \to x_i} \tag{3}$$

Others properties

$$
\begin{aligned}
\int_{-\infty}^{\infty} N(x|\mu_x,\sigma_x^2)N(x|\mu_y,\sigma_y^2)\,dx &\overset{*}{=} \int_{-\infty}^{\infty} \underbrace{N(\mu_x|\mu_y,\sigma_x^2+\sigma_y^2)}_{\text{constante}} N(x|\mu_*,\sigma_*^2)dx \\
&= N(\mu_x|\mu_y,\sigma_x^2+\sigma_y^2) \underbrace{\int_{-\infty}^{\infty} N(x|\mu_*,\sigma_*^2)dx}_{1} \\
&= N(\mu_x|\mu_y,\sigma_x^2+\sigma_y^2)
\end{aligned} \tag{4}
$$

where the equality indicated ($\overset{*}{=}$) is developed in section Normal product.

$$
\begin{aligned}
\int_{-\infty}^{\infty}\int_{-\infty}^{\infty} \mathbb{I}(x=h(y,z))f(x)g(y)\,dx\,dy &= \int_{-\infty}^{\infty}\int_{h(y,z)}^{h(y,z)} f(h(y,z))g(y)\,dx\,dy \\
&= \int_{-\infty}^{\infty} f(h(y,z))g(y)dy
\end{aligned} \tag{5}
$$

$$
N(x|\mu,\sigma^2) = N(\mu|x,\sigma^2) = N(-\mu|-x,\sigma^2) = N(-x|-\mu,\sigma^2) \tag{6}
$$

$$
\frac{\partial}{\partial x}\Phi(x|\mu,\sigma^2) = N(x|\mu,\sigma^2) \tag{7}
$$

$$
X \sim N(\mu,\sigma^2) \Rightarrow \frac{X-\mu}{\sigma} \sim N(0,1) \tag{8}
$$

Example (2 vs 2) Two teams with two members.

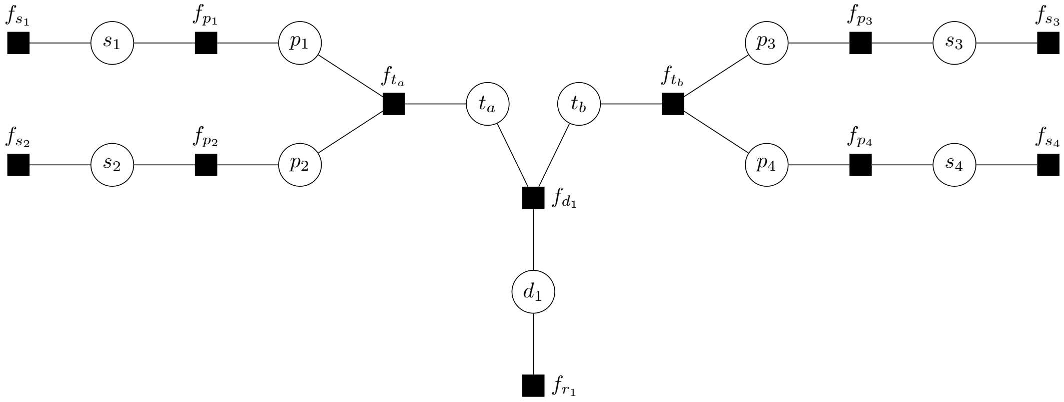

$fs_i = N(s_i;\mu_i,\sigma^2)$ $fp_i = N(p_i;s_i,\beta^2)$ $ft_e = \mathbb{I}(t_e = \sum_{i\in A_e} p_i)$ $fd_k = \mathbb{I}(d_k = t_{(k)} - t_{(k+1)})$ $fr_k = \mathbb{I}(d_k > 0)$

Fig B. Factor graph of TrueSkill, 2 vs 2

$\boldsymbol{m_{f_s\to s}(s)}$:

$$
\begin{aligned}
m_{f_{s_i}\to s_i}(s_i) &\overset{\text{eq}}{\underset{2}{=}} \int\cdots\int f_{s_i}(\boldsymbol{x}) \prod_{h\in n(f_{s_i})\setminus\{s_i\}} m_{h\to f_{s_i}}(h)d\boldsymbol{x}_{\setminus\{s_i\}} \\
&\overset{\text{fig}}{\underset{B}{=}} \int\cdots\int N(s_i|\mu_i,\sigma_i^2)d\boldsymbol{x}_{\setminus\{s_i\}} \\
&\overset{\text{eq}}{\underset{2}{=}} N(s_i|\mu_i,\sigma_i^2)
\end{aligned} \tag{9}
$$

$\boldsymbol{m_{s \rightarrow f_p}(s)}$:

$$m_{s_i \rightarrow f_{p_i}}(s_i) \overset{\underset{1}{\text{eq}}}{=} \prod_{g \in n(s_i) \setminus \{f_{p_i}\}} m_{g \rightarrow s_i}(s_i) \overset{\underset{B}{\text{fig}}}{=} m_{f_{s_i} \rightarrow s_i}(s_i) \overset{\underset{9}{\text{eq}}}{=} N(s_i|\mu_i, \sigma_i^2) \tag{10}$$

$\boldsymbol{m_{f_p \rightarrow p}(p)}$:

$$\begin{aligned} m_{f_{p_i} \rightarrow p_i}(p_i) &\overset{\underset{2}{\text{eq}}}{=} \int \cdots \int f_{p_i}(\boldsymbol{x}) \prod_{h \in n(f_{p_i}) \setminus \{p_i\}} m_{h \rightarrow f_{p_i}}(h) d\boldsymbol{x}_{\setminus \{p_i\}} \\ &\overset{\underset{B}{\text{fig}}}{\underset{\underset{10}{\text{eq}}}{=}} \int \cdots \int N(p_i|s_i, \beta^2) N(s_i|\mu_i, \sigma_i^2) d\boldsymbol{x}_{\setminus \{p_i\}} \\ &\overset{\underset{2}{\text{eq}}}{=} \int N(p_i|s_i, \beta^2) N(s_i|\mu_i, \sigma_i^2) ds_i \\ &\overset{\underset{6}{\text{eq}}}{=} \int N(s_i|p_i, \beta^2) N(s_i|\mu_i, \sigma_i^2) ds_i \\ &\overset{\underset{4}{\text{eq}}}{=} N(p_i|\mu_i, \beta^2 + \sigma_i^2) \end{aligned} \tag{11}$$

$\boldsymbol{m_{p \rightarrow f_t}(p)}$:

$$\begin{aligned} m_{p_i \rightarrow f_{t_e}}(p_i) &\overset{\underset{1}{\text{eq}}}{=} \prod_{g \in n(p_i) \setminus \{f_{t_e}\}} m_{g \rightarrow p_i}(p_i) \\ &\overset{\underset{B}{\text{fig}}}{=} m_{f_{p_i} \rightarrow p_i}(p_i) \overset{\underset{11}{\text{eq}}}{=} N(p_i|\mu_i, \beta^2 + \sigma_i^2) \end{aligned} \tag{12}$$

$\boldsymbol{m_{f_t \rightarrow t}(t)}$:

$$\begin{aligned} m_{f_{t_e} \rightarrow t_e}(t_e) &\overset{\underset{2}{\text{eq}}}{=} \int \cdots \int f_{t_e}(\boldsymbol{x}) \prod_{h \in n(f_{t_e}) \setminus \{t_e\}} m_{h \rightarrow f_{t_e}}(h) d\boldsymbol{x}_{\setminus \{t_e\}} \\ &\overset{\underset{B}{\text{fig}}}{\underset{\underset{12}{\text{eq}}}{=}} \int \cdots \int \mathbb{I}(t_e = p_i + p_j) N(p_i|\mu_i, \beta^2 + \sigma_i^2) N(p_j|\mu_j, \beta^2 + \sigma_j^2) d\boldsymbol{x}_{\setminus \{t_e\}} \\ &\overset{\underset{2}{\text{eq}}}{=} \iint \mathbb{I}(t_e = p_i + p_j) N(p_i|\mu_i, \beta^2 + \sigma_i^2) N(p_j|\mu_j, \beta^2 + \sigma_j^2) dp_i dp_j \\ &\overset{\underset{5}{\text{eq}}}{=} \int N(p_i|\mu_i, \beta^2 + \sigma_i^2) N(t_e - p_i|\mu_j, \beta^2 + \sigma_j^2) dp_i \\ &\overset{\underset{6}{\text{eq}}}{=} \int N(p_i|\mu_i, \beta^2 + \sigma_i^2) N(p_i|t_e - \mu_j, \beta^2 + \sigma_j^2) dp_i \\ &\overset{\underset{4}{\text{eq}}}{=} N(t_e|\mu_i + \mu_j, 2\beta^2 + \sigma_i^2 + \sigma_j^2) \end{aligned} \tag{13}$$

General (by induction)

$$m_{f_{t_e} \rightarrow t_e}(t_e) = N\Big(t_e | \underbrace{\sum_{i \in A_e} \mu_i}_{\substack{\text{Team} \\ \text{skill}} \ \mu_e}, \underbrace{\sum_{i \in A_e} \beta^2 + \sigma_i^2}_{\substack{\text{Team} \\ \text{variance}} \ \sigma_e^2}\Big) = N(t_e|\mu_e, \sigma_e^2) \tag{14}$$

$\boldsymbol{m_{t\rightarrow f_d}(t)}$:

$$m_{t_e\rightarrow f_{d_k}}(d_k) \overset{\text{eq}}{\underset{1}{=}} \prod_{g\in n(t_e)\setminus\{f_{d_k}\}} m_{g\rightarrow t_e}(t_e) \\ \overset{\text{fig}}{\underset{B}{=}} m_{f_{t_e}\rightarrow t_e}(t_e) \overset{\text{eq}}{\underset{14}{=}} N(t_e|\sum_{i\in A_e}\mu_i, \sum_{i\in A_e}\beta^2+\sigma_i^2) \overset{\text{eq}}{\underset{14}{=}} N(t_e|\mu_e,\sigma_e^2) \tag{15}$$

$\boldsymbol{m_{f_d\rightarrow d}(d)}$:

$$\begin{aligned} m_{f_{d_1}\rightarrow d_1}(d_1) &\overset{\text{eq}}{\underset{2}{=}} \int\cdots\int f_{d_1}(\boldsymbol{x}) \prod_{h\in n(f_{d_1})\setminus\{d_1\}} m_{h\rightarrow f_{d_1}}(h)\, d\boldsymbol{x}_{\setminus\{d_1\}} \\ &\underset{\substack{\text{eq}\\15}}{\overset{\substack{\text{fig}\\B}}{=}} \int\int \mathbb{I}(d_1=t_a-t_b)N(t_a|\mu_a,\sigma_a^2)N(t_b|\mu_b,\sigma_b^2)dt_a dt_b \\ &\overset{\text{eq}}{\underset{5}{=}} \int N(d_1+t_b|\mu_a,\sigma_a^2)N(t_b|\mu_a,\sigma_b^2)dt_b \\ &\overset{\text{eq}}{\underset{6}{=}} \int N(t_b|\mu_a-d_1,\sigma_a^2)N(t_b|\mu_b,\sigma_b^2)dt_b \\ &\overset{\text{eq}}{\underset{4}{=}} N(\mu_a-d_1|\mu_b,\sigma_a^2+\sigma_b^2) \\ &\overset{\text{eq}}{\underset{6}{=}} N(d_1|\mu_a-\mu_b,\sigma_a^2+\sigma_b^2) \end{aligned} \tag{16}$$

General

$$m_{f_{d_1}\rightarrow d_1}(d_1) = N\Bigg(d_1 \mid \underbrace{\sum_{i\in A_a}\mu_i - \sum_{i\in A_b}\mu_i}_{\substack{\text{Expected}\\\text{difference}}\ (\delta_1)}\ ,\ \underbrace{\sum_{i\in A_a\cup A_b}\beta^2+\sigma_i^2}_{\substack{\text{Total}\\\text{variance}}\ (\vartheta_1)}\Bigg) = N(d_1|\delta_1,\vartheta_1) \tag{17}$$

$\boldsymbol{m_{f_r\rightarrow d}(d)}$:

$$m_{f_r\rightarrow d_1}(d_1) \underset{\substack{\text{eq}\\2}}{\overset{\substack{\text{fig}\\B}}{=}} \mathbb{I}(d_1>0) \tag{18}$$

$\boldsymbol{m_{f_{d_1}\rightarrow t_a}(t_a)}$: (Winning case)

$$\begin{aligned} m_{f_{d_1}\rightarrow t_a}(t_a) &\overset{\text{eq}}{\underset{2}{=}} \int\cdots\int f_{d_1}(\boldsymbol{x}) \prod_{h\in n(f_{d_1})\setminus\{t_a\}} m_{h\rightarrow f_{d_1}}(h)\, d\boldsymbol{x}_{\setminus\{t_a\}} \\ &\underset{\substack{\text{eq}\\15}}{\overset{\substack{\text{fig}\\B}}{=}} \int\cdots\int \mathbb{I}(d_1=t_a-t_b)\mathbb{I}(d_1>0)N(t_b|\mu_b,\sigma_b^2)\, d\boldsymbol{x}_{\setminus\{t_a\}} \\ &\overset{\text{eq}}{\underset{2}{=}} \iint \mathbb{I}(d_1=t_a-t_b)\mathbb{I}(d_1>0)N(t_b|\mu_b,\sigma_b^2)\, dd_1\, dt_b \\ &\overset{\text{eq}}{\underset{5}{=}} \int \mathbb{I}(t_a>t_b)N(t_b|\mu_b,\sigma_b^2)\, dt_b \\ &\overset{\text{fig}}{\underset{C}{=}} \Phi(t_a|\mu_b,\sigma_b^2) \overset{\substack{\mu_b\\\sigma_b}}{=} \Phi\Big(t_a|\sum_{i\in A_b}\mu_i, \sum_{i\in A_b}\beta^2+\sigma_i^2\Big) \end{aligned} \tag{19}$$

$\boldsymbol{m_{f_{d_1}\to t_b}(t_b)}$: (Loosing case)

$$\begin{aligned}
m_{f_{d_1}\to t_b}(t_b) &\overset{\substack{\text{eq}\\2}}{=} \int\cdots\int f_{d_1}(\boldsymbol{x}) \prod_{h\in n(f_{d_1})\setminus\{t_b\}} m_{h\to f_{d_1}}(h)\, d\boldsymbol{x}_{\setminus\{t_a\}} \\
&\overset{\substack{\text{fig}\\B}}{\underset{\substack{\text{eq}\\15}}{=}} \int\cdots\int \mathbb{I}(d_1 = t_a - t_b)\mathbb{I}(d_1 > 0) N(t_a|\mu_a, \sigma_a^2)\, d\boldsymbol{x}_{\setminus\{t_b\}} \\
&\overset{\substack{\text{eq}\\2}}{=} \iint \mathbb{I}(d_1 = t_a - t_b)\mathbb{I}(d_1 > 0) N(t_a|\mu_a, \sigma_a^2)\, dd_1\, dt_a \\
&\overset{\substack{\text{eq}\\5}}{=} \int \mathbb{I}(t_a > t_b) N(t_a|\mu_a, \sigma_a^2)\, dt_a \\
&\overset{\substack{\text{fig}\\C}}{=} 1 - \Phi(t_b|\mu_a, \sigma_a^2) \overset{\substack{\mu_a\\\sigma_a}}{=} 1 - \Phi\Big(t_b|\sum_{i\in A_a}\mu_i, \sum_{i\in A_a}\beta^2 + \sigma_i^2\Big)
\end{aligned} \tag{20}$$

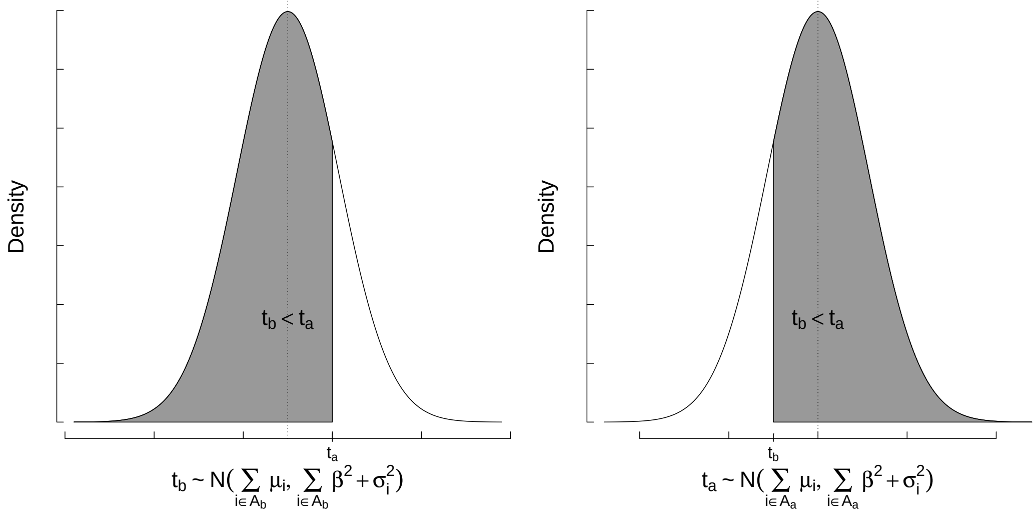

(a) $m_{f_{d_1}\to t_a}(t_a)$ (Winning case) **(b)** $m_{f_{d_1}\to t_b}(t_b)$ (Loosing case)

Fig C

$\boldsymbol{m_{t_a\to f_{t_a}}(t_a)}$: (Winning case)

$$m_{t_a\to f_{t_a}}(t_a) \overset{\substack{\text{eq}\\1}}{=} \prod_{g\in n(t_a)\setminus\{f_{t_a}\}} m_{g\to t_a}(t_a) \overset{\substack{\text{eq}\\19}}{=} \Phi(t_a|\mu_b, \sigma_b^2) \overset{\substack{\mu_b\\\sigma_b}}{=} \Phi\Big(t_a|\sum_{i\in A_b}\mu_i, \sum_{i\in A_b}\beta^2 + \sigma_i^2\Big) \tag{21}$$

$\boldsymbol{m_{t_b\to f_{t_b}}(t_b)}$: (Loosing case)

$$m_{t_b\to f_{t_b}}(t_b) \overset{\substack{\text{eq}\\1}}{=} \prod_{g\in n(t_b)\setminus\{f_{t_b}\}} m_{g\to t_b}(t_b) \overset{\substack{\text{eq}\\20}}{=} 1 - \Phi(t_b|\mu_a, \sigma_a^2) \overset{\substack{\mu_a\\\sigma_a}}{=} 1 - \Phi\Big(t_b|\sum_{i\in A_a}\mu_i, \sum_{i\in A_a}\beta^2 + \sigma_i^2\Big) \tag{22}$$

$\boldsymbol{m_{f_{t_a}\to p_1}(p_1)}$: (Winning case)

$$
\begin{aligned}
m_{f_{t_a}\to p_1}(p_1) &\overset{\text{eq}}{\underset{2}{=}} \int\cdots\int f_{t_a}(\boldsymbol{x}) \prod_{h\in n(f_{t_a})\setminus\{p_1\}} m_{h\to f_{t_a}}(h)\, d\boldsymbol{x}_{\setminus\{p_1\}} \\
&\overset{\substack{\text{fig}\\ B}}{\underset{\substack{\text{eq}\\ 21}}{=}} \int\cdots\int \mathbb{I}(t_a = p_1 + p_2) N(p_2|\mu_2, \beta^2 + \sigma_2^2)\, \Phi(t_a|\mu_b, \sigma_b^2)\, d\boldsymbol{x}_{\setminus\{p_1\}} \\
&\overset{\text{eq}}{\underset{2}{=}} \iint \mathbb{I}(t_a = p_1 + p_2)\, N(p_2|\mu_2, \beta^2 + \sigma_2^2)\, \Phi(t_a|\mu_b, \sigma_b^2)\, dt_a dp_2 \\
&\overset{\text{eq}}{\underset{5}{=}} \int N(p_2|\mu_2, \beta^2 + \sigma_2^2)\, \Phi(p_1 + p_2|\mu_b, \sigma_b^2)\, dp_2 \\
&\overset{\text{eq}}{\underset{6}{=}} \int N(p_2|\mu_2, \beta^2 + \sigma_2^2)\, \Phi(p_1|\mu_b - p_2, \sigma_b^2)\, dp_2 \\
&= \kappa(p_1)
\end{aligned} \tag{23}
$$

Knowing that $\partial\Phi(\,) = N(\,)$, we continue as follows.

$$
\begin{aligned}
\frac{\partial\kappa(x)}{\partial x} &= \frac{\partial}{\partial x} \int N(y|\mu_y, \sigma_y^2)\, \Phi(x|\mu_x - y, \sigma_x^2)\, dy \\
&= \int N(y|\mu_y, \sigma_y^2)\, \frac{\partial}{\partial x}\, \Phi(x|\mu_x - y, \sigma_x^2)\, dy \\
&\overset{\text{eq}}{\underset{7}{=}} \int N(y|\mu_y, \sigma_y^2)\, N(x|\mu_x - y, \sigma_x^2)\, dy \\
&\overset{\text{eq}}{\underset{6}{=}} \int N(y|\mu_y, \sigma_y^2)\, N(y|\mu_x - x, \sigma_x^2)\, dy \\
&\overset{\substack{\text{eq}\\ 4}}{\underset{\substack{\text{eq}\\ 6}}{=}} N(x|\mu_x - \mu_y, \sigma_x^2 + \sigma_y^2)
\end{aligned} \tag{24}
$$

Then,

$$
m_{f_{t_a}\to p_1}(p_1) \overset{\substack{\text{eq}\\ 23}}{\underset{\substack{\text{eq}\\ 24}}{=}} \Phi(p_1|\mu_b - \mu_2, \beta^2 + \sigma_2^2 + \sigma_b^2) \overset{\substack{\mu_b\\ \sigma_b}}{=} \Phi\Big(p_1|\sum_{i\in A_b} \mu_i - \mu_2, \beta^2 + \sigma_2^2 + \sum_{i\in A_b} \beta^2 + \sigma_i^2\Big) \tag{25}
$$

$\boldsymbol{m_{f_{t_b}\to p_3}(p_3)}:$ (Loosing case)

$$
\begin{aligned}
m_{f_{t_b}\to p_3}(p_3) &\overset{\text{eq}}{\underset{2}{=}} \int\cdots\int f_{t_a}(\boldsymbol{x}) \prod_{h\in n(f_{t_a})\setminus\{p_1\}} m_{h\to f_{t_a}}(h)\, d\boldsymbol{x}_{\setminus\{p_1\}} \\
&\overset{\substack{\text{fig}\\B}}{\underset{\substack{\text{eq}\\22}}{=}} \int\cdots\int \mathbb{I}(t_b = p_3 + p_4)\,(1-\Phi(t_b|\mu_a,\sigma_a^2))\,N(p_4|\mu_4,\beta^2+\sigma_4^2)\,d\boldsymbol{x}_{\setminus\{p_3\}} \\
&\overset{\text{eq}}{\underset{2}{=}} \iint \mathbb{I}(t_b = p_3 + p_4) N(p_4|\mu_4,\beta^2+\sigma_4^2)(1-\Phi(t_b|\mu_a,\sigma_a^2))\,dt_b dp_4 \\
&\overset{\substack{\text{eq}\\5}}{\underset{\substack{\text{eq}\\6}}{=}} \int N(p_4|\mu_4,\beta^2+\sigma_4^2)(1-\Phi(p_3|\mu_a - p_4,\sigma_a^2))\,dp_4 \\
&= \underbrace{\int N(p_4|\mu_4,\beta^2+\sigma_4^2)dp_4}_{1} - \underbrace{\int N(p_4|\mu_4,\beta^2+\sigma_4^2)\Phi(p_3|\mu_a - p_4,\sigma_a^2))\,dp_4}_{\kappa(p_3)} \\
&\overset{\text{eq}}{\underset{24}{=}} 1-\Phi(p_3,\mu_a-\mu_4,\beta^2+\sigma_4^2+\sigma_a^2) \\
&\overset{\mu_a}{\underset{\sigma_a}{=}} 1-\Phi\Big(p_3, \sum_{i\in A_a}\mu_i - \mu_4, \beta^2+\sigma_4^2+\sum_{i\in A_a}\beta^2+\sigma_i^2\Big)
\end{aligned}
\tag{26}
$$

$\boldsymbol{m_{p_1\to f_{p_1}}(s_1)}:$ (Winning case)

$$
m_{p_1\to f_{p_1}}(p_1) \overset{\text{eq}}{\underset{1}{=}} \prod_{g\in n(p_1)\setminus\{f_{p_1}\}} m_{g\to p_1}(p_1) \overset{\text{eq}}{\underset{25}{=}} \Phi(p_1|\mu_b-\mu_2,\beta^2+\sigma_2^2+\sigma_b^2)
\tag{27}
$$

$\boldsymbol{m_{p_3\to f_{p_3}}(s_1)}:$ (Loosing case)

$$
m_{p_3\to f_{p_3}}(p_3) \overset{\text{eq}}{\underset{1}{=}} \prod_{g\in n(p_3)\setminus\{f_{p_3}\}} m_{g\to p_3}(p_3) \overset{\text{eq}}{\underset{26}{=}} 1-\Phi(p_3,\mu_a-\mu_4,\beta^2+\sigma_4^2+\sigma_a^2)
\tag{28}
$$

$\boldsymbol{m_{f_{p_1}\to s_1}(s_1)}:$ (Winning case)

$$
\begin{aligned}
m_{f_{p_1}\to s_1}(s_1) &\overset{\text{eq}}{\underset{2}{=}} \int\cdots\int f_{p_1}(\boldsymbol{x}) \prod_{h\in n(f_{p_1})\setminus\{s_1\}} m_{h\to f_{p_1}}(h)\, d\boldsymbol{x}_{\setminus\{s_1\}} \\
&\overset{\substack{\text{fig}\\B}}{\underset{\substack{\text{eq}\\27}}{=}} \int\cdots\int N(p_1|s_1,\beta^2)\,\Phi(p_1|\mu_b-\mu_2,\beta^2+\sigma_2^2+\sigma_b^2)\,d\boldsymbol{x}_{\setminus\{s_1\}} \\
&\overset{\substack{\text{eq}\\2}}{\underset{\substack{\text{eq}\\6}}{=}} \int N(p_1|s_1,\beta^2)\,\Phi(\mu_2|\mu_b-p_1,\beta^2+\sigma_2^2+\sigma_b^2)\,dp_1 \\
&\overset{\text{eq}}{\underset{24}{=}} \Phi(s_1|\mu_b-\mu_2,2\beta^2+\sigma_2^2+\sigma_b^2)
\end{aligned}
\tag{29}
$$

General (N vs N)

$$\begin{aligned} m_{f_{p_1}\to s_1}(s_1) &\overset{\text{eq}}{\underset{29}{=}} \Phi(s_1|\mu_b - \mu_a + \mu_1, \sigma_b^2 + \sigma_a^2 - \sigma_1^2) \\ &\overset{\mu_b}{\underset{\sigma_b}{=}} \Phi\Big(s_1| \underbrace{\sum_{i\in A_b}\mu_i - \sum_{i\in A_a}\mu_i}_{-\text{ Expected difference } -\delta_{ab}=\delta_{ba}} + \mu_1, \underbrace{\sum_{i\in A_b\cup A_a}\beta^2 + \sigma_i^2}_{\text{Total variance } \vartheta^2} - \sigma_1^2\Big) \\ &\overset{\delta}{\underset{\vartheta}{=}} \Phi(s_1| - \delta_{ab} + \mu_1, \vartheta^2 - \sigma_1^2) \\ &\overset{\text{eq}}{\underset{6}{=}} 1 - \Phi(0| \underbrace{\delta_{ab} - \mu_1 + s_1}_{\text{Parameterized expected difference } \delta_1(s_1)}, \underbrace{\vartheta^2 - \sigma_1^2}_{\vartheta_1^2}) \\ &\overset{\delta_1}{\underset{\vartheta_1}{=}} 1 - \Phi(0|\delta_1(s_1), \vartheta_1^2) \\ &\overset{\text{eq}}{\underset{8}{=}} 1 - \Phi\Big(\frac{0 - \delta_1(s_1)}{\vartheta_1}\Big) \\ &\overset{\text{eq}}{\underset{6}{=}} \Phi\Big(\frac{\delta_1(s_1)}{\vartheta_1}\Big) \end{aligned} \tag{30}$$

Note: the message $m_{f_{p_1}\to s_1}(s_1)$ compute the "Parameterized winning probability", i.e. the winning probability knowing the player skill. If we know the player skill, we must use the true skill to compute the mean of the belief distribution (what we do in $\delta_1(s_1)$), eliminating therefore their variance from the total variance (what we do in ϑ_1).

$\boldsymbol{m_{f_{p_3}\to s_3}(s_3)}$: (Loosing case)

$$\begin{aligned} m_{f_{p_3}\to s_3}(s_3) &\overset{\text{eq}}{\underset{2}{=}} \int \cdots \int f_{p_3}(\boldsymbol{x}) \prod_{h\in n(f_{p_3})\setminus\{s_3\}} m_{h\to f_{p_3}}(h)\, d\boldsymbol{x}_{\setminus\{s_3\}} \\ &\overset{\text{fig}}{\underset{\substack{B \\ \text{eq} \\ 28}}{=}} \int \cdots \int N(p_3|s_3, \beta^2)(1 - \Phi(p_3, \mu_a - \mu_4, \beta^2 + \sigma_4^2 + \sigma_a^2))\, d\boldsymbol{x}_{\setminus\{s_3\}} \\ &\overset{\text{eq}}{\underset{\substack{2 \\ \text{eq} \\ 6}}{=}} \int N(p_3|s_3, \beta^2)(1 - \Phi(\mu_4, \mu_a - p_3, \beta^2 + \sigma_4^2 + \sigma_a^2))\, dp_3 \\ &= \int N(p_3|s_3, \beta^2)\, dp_3 - \int N(p_3|s_3, \beta^2)\Phi(\mu_4, \mu_a - p_3, \beta^2 + \sigma_4^2 + \sigma_a^2)\, dp_3 \\ &\overset{\text{eq}}{\underset{24}{=}} 1 - \Phi\Big(s_3|\mu_a - \mu_4, 2\beta^2 + \sigma_4^2 + \sigma_a^2\Big) \end{aligned} \tag{31}$$

General (N vs N)

$$\begin{aligned} m_{f_{p_3}\to s_3}(s_3) &\overset{\text{eq}}{\underset{31}{=}} 1 - \Phi\Big(s_3| \underbrace{\mu_a - \mu_b}_{-\delta_{ba}} + \mu_3, \underbrace{\sigma_a^2 + \sigma_b^2}_{\vartheta^2} - \sigma_3^2\Big) \\ &\overset{\delta}{\underset{\vartheta}{=}} 1 - \Phi\Big(s_3| - \delta_{ba} + \mu_3, \vartheta^2 - \sigma_3^2\Big) \overset{\text{eq}}{\underset{6}{=}} \Phi\Big(0| \underbrace{\delta_{ba} - \mu_3 + s_3}_{\delta_3(s_3)}, \underbrace{\vartheta^2 - \sigma_3^2}_{\vartheta_3^2}\Big) \\ &\overset{\delta_3}{\underset{\vartheta_3}{=}} \Phi(0|\delta_3(s_3), \vartheta_3^2) \overset{\text{eq}}{\underset{8}{=}} \Phi\left(\frac{0 - \delta_3(s_3)}{\vartheta_3}\right) \\ &\overset{\text{eq}}{\underset{6}{=}} \Phi\Big(\frac{-\delta_3(s_3)}{\vartheta_3}\Big) \end{aligned} \tag{32}$$

Note: the message $m_{f_{p_3} \to s_3}(s_1)$ compute the "Parameterized loosing probability", i.e. the loosing probability knowing the player skill. If we know the player skill, we must use the true skill to compute the mean of the belief distribution (what we do in $\delta_3(s_3)$), eliminating therefore their variance from the total variance (what we do in ϑ_3).

Posterior: winner case

$$p(s_1|o,A) \overset{\text{eq}}{\underset{3}{=}} \prod_{h \in n(x_i)} m_{h \to x_i} \overset{\text{fig}}{\underset{\substack{\text{eq}\\30}}{\overset{B}{=}}} N(s_1|\mu_1,\sigma_1^2)\Phi\left(\frac{\delta_1(s_1)}{\vartheta_1}\right) \tag{33}$$

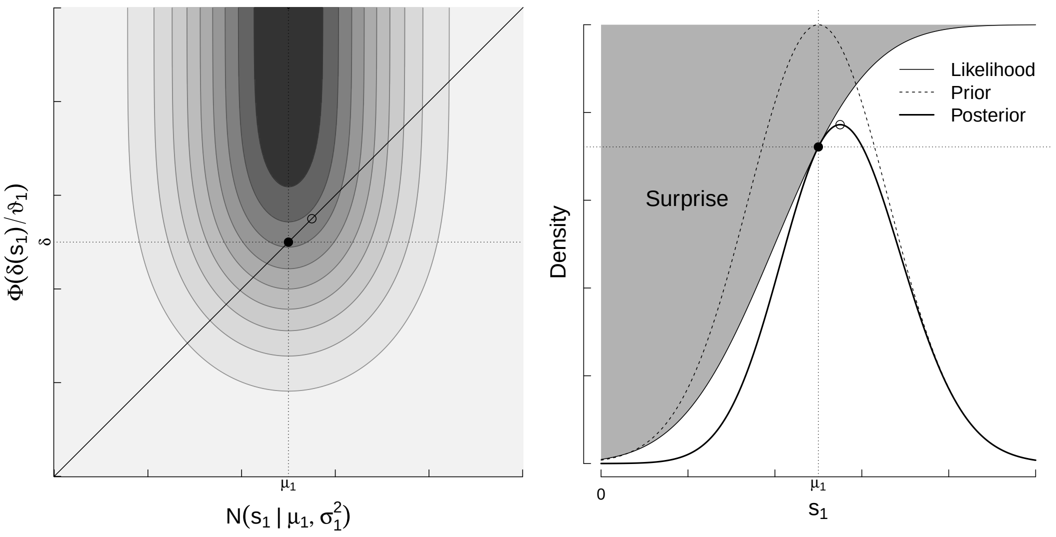

(a) **(b)**

Fig D. Winner exact posterior

Posterior: looser case

$$p(s_1|o,A) \overset{\text{eq}}{\underset{3}{=}} \prod_{h \in n(x_i)} m_{h \to x_i} \overset{\text{fig}}{\underset{\substack{\text{eq}\\30}}{\overset{B}{=}}} N(s_1|\mu_1,\sigma_1^2)\Phi\left(\frac{-\delta_1(s_1)}{\vartheta_1}\right) \tag{34}$$

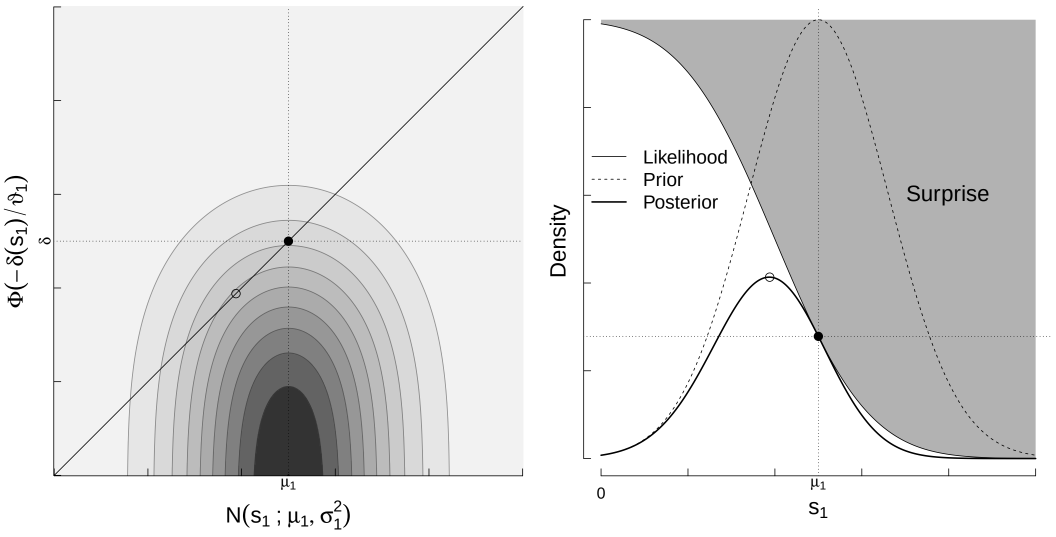

(a) (b)

Fig E. Looser exact posterior

Normal product

The problem that we have to solve is

$$\int N(x;\mu_1,\sigma_1^2)N(x;\mu_2,\sigma_2^2)dx \tag{35}$$

By definition,

$$\begin{aligned} N(x;y,\beta^2)N(x;\mu,\sigma^2) &= \frac{1}{\sqrt{2\pi}\sigma_1}e^{-\frac{(x-\mu_1)^2}{2\sigma_1^2}}\frac{1}{\sqrt{2\pi}\sigma_2}e^{-\frac{(x-\mu_2)^2}{2\sigma_2^2}} \\ &= \frac{1}{2\pi\sigma_1\sigma_2}\exp\Bigg(-\underbrace{\left(\frac{(x-\mu_1)^2}{2\sigma_1^2}+\frac{(x-\mu_2)^2}{2\sigma_2^2}\right)}_{\theta}\Bigg) \end{aligned} \tag{36}$$

Then,

$$\theta = \frac{\sigma_2^2(x^2+\mu_1^2-2x\mu_1)+\sigma_1^2(x^2+\mu_2^2-2x\mu_2)}{2\sigma_1^2\sigma_2^2} \tag{37}$$

Expanding and reordering the factors by powers of x

$$\frac{(\sigma_1^2+\sigma_2^2)x^2-(2\mu_1\sigma_2^2+2\mu_2\sigma_1^2)x+(\mu_1^2\sigma_2^2+\mu_2^2\sigma_1^2)}{2\sigma_1^2\sigma_2^2} \tag{38}$$

We divide the numerator and the denominator by the factor of x^2

$$\frac{x^2-2\frac{(\mu_1\sigma_2^2+\mu_2\sigma_1^2)}{(\sigma_1^2+\sigma_2^2)}x+\frac{(\mu_1^2\sigma_2^2+\mu_2^2\sigma_1^2)}{(\sigma_1^2+\sigma_2^2)}}{2\frac{\sigma_1^2\sigma_2^2}{(\sigma_1^2+\sigma_2^2)}} \tag{39}$$

This equation is quadratic in x, and therefore is proportional to a Gaussian density function with standard deviation,

$$\sigma_\times = \sqrt{\frac{\sigma_1^2\sigma_2^2}{\sigma_1^2+\sigma_2^2}} \tag{40}$$

and mean

$$\mu_\times = \frac{(\mu_1\sigma_2^2 + \mu_2\sigma_1^2)}{(\sigma_1^2 + \sigma_2^2)} \tag{41}$$

Since $\varepsilon = 0$ can be added to complete the square in θ, this test is enough when a normalization is not needed. Let,

$$\varepsilon = \frac{\mu_\times^2 - \mu_\times^2}{2\sigma_\times^2} = 0 \tag{42}$$

$$\theta = \frac{x^2 - 2\mu_\times x + \mu_\times^2}{2\sigma_\times^2} + \underbrace{\frac{\frac{(\mu_1^2\sigma_2^2+\mu_2^2\sigma_1^2)}{(\sigma_1^2+\sigma_2^2)} - \mu_\times^2}{2\sigma_\times^2}}_{\varphi} \tag{43}$$

Reordering φ

$$\begin{aligned}\varphi &= \frac{\frac{(\mu_1^2\sigma_2^2+\mu_2^2\sigma_1^2)}{(\sigma_1^2+\sigma_2^2)} - \left(\frac{(\mu_1\sigma_2^2+\mu_2\sigma_1^2)}{(\sigma_1^2+\sigma_2^2)}\right)^2}{2\frac{\sigma_1^2\sigma_2^2}{\sigma_1^2+\sigma_2^2}} \\ &= \frac{(\sigma_1^2+\sigma_2^2)(\mu_1^2\sigma_2^2+\mu_2^2\sigma_1^2) - (\mu_1\sigma_2^2+\mu_2\sigma_1^2)^2}{\sigma_1^2+\sigma_2^2}\frac{1}{2\sigma_1^2\sigma_2^2} \\ &= \frac{(\mu_1^2\sigma_1^2\sigma_2^2 + \cancel{\mu_2^2\sigma_1^4} + \cancel{\mu_1^2\sigma_2^4} + \mu_2^2\sigma_1^2\sigma_2^2) - (\cancel{\mu_1^2\sigma_2^4} + 2\mu_1\mu_2\sigma_1^2\sigma_2^2 + \cancel{\mu_2^2\sigma_1^4})}{\sigma_1^2+\sigma_2^2}\frac{1}{2\sigma_1^2\sigma_2^2}\end{aligned} \tag{44}$$

$$\theta = \frac{(x-\mu_\times)}{2\sigma_\times^2} + \frac{(\mu_1-\mu_2)^2}{2(\sigma_1^2+\sigma_2^2)} \tag{45}$$

Then,

$$\begin{aligned}N(x; y, \beta^2)N(x; \mu, \sigma^2) &= \frac{1}{2\pi\sigma_1\sigma_2}\exp\Bigg(-\underbrace{\left(\frac{(x-\mu_\times)}{2\sigma_\times^2} + \frac{(\mu_1-\mu_2)^2}{2(\sigma_1^2+\sigma_2^2)}\right)}_{\theta}\Bigg) \\ &= \frac{1}{2\pi\sigma_1\sigma_2}\exp\left(-\frac{(x-\mu_\times)}{2\sigma_\times^2}\right)\exp\left(-\frac{(\mu_1-\mu_2)^2}{2(\sigma_1^2+\sigma_2^2)}\right)\end{aligned} \tag{46}$$

Multiplying by $\sigma_\times\sigma_\times^{-1}$

$$\overbrace{\frac{\cancel{\sigma_1\sigma_2}}{\sqrt{\sigma_1^2+\sigma_2^2}}}^{\sigma_\times}\frac{1}{\sigma_\times}\frac{1}{2\pi\cancel{\sigma_1\sigma_2}}\exp\left(-\frac{(x-\mu_\times)}{2\sigma_\times^2}\right)\exp\left(-\frac{(\mu_1-\mu_2)^2}{2(\sigma_1^2+\sigma_2^2)}\right) \tag{47}$$

Then,

$$\frac{1}{\sqrt{2\pi}\sigma_\times}\exp\left(-\frac{(x-\mu_\times)}{2\sigma_\times^2}\right)\frac{1}{\sqrt{2\pi(\sigma_1^2+\sigma_2^2)}}\exp\left(-\frac{(\mu_1-\mu_2)^2}{2(\sigma_1^2+\sigma_2^2)}\right) \tag{48}$$

Going back to the integral,

$$\begin{aligned}I &= \int N(x; \mu_\times, \sigma_\times^2)\overbrace{N(\mu_1; \mu_2, \sigma_1^2+\sigma_2^2)}^{\text{Scalar independent of x}}\,dx \\ &= N(\mu_1; \mu_2, \sigma_1^2+\sigma_2^2)\underbrace{\int N(x, \mu_\times, \sigma_\times^2)dx}_{\text{Integrate 1}} \\ &= N(\mu_1; \mu_2, \sigma_1^2+\sigma_2^2)\end{aligned} \tag{49}$$

Sum of n Gaussian

By induction.

$$P(n) := \sum_{i=1}^{n} x_i \sim N(\sum_{i=1}^{n} \mu_i, \sigma_i^2) \tag{50}$$

Base case

$$P(1) := \int \mathbb{I}(t_1 = x_1) N(x_1; \mu_1, \sigma_1^2) dx_1 = N(x; \mu_1, \sigma_1^2) \tag{51}$$

Then $P(1)$ is true.

$$\begin{aligned} P(2) &:= \iint \mathbb{I}(t_2 = x_1 + x_2) N(x_1|\mu_1, \sigma_1^2) N(x_2|\mu_2, \sigma_2^2) dx_1 dx_2 \\ &= \int N(x_1|\mu_1, \sigma_1^2) N(t_2 - x_1|\mu_2, \sigma_2^2) dx_1 \\ &= \int N(x_1|\mu_1, \sigma_1^2) N(x_1|t_2 - \mu_2, \sigma_2^2) dx_1 \\ &\stackrel{*}{=} \int \underbrace{N(t_2|\mu_1 + \mu_2, \sigma_1^2 + \sigma_2^2)}_{\text{const.}} \underbrace{N(x_1|\mu_*, \sigma_*^2) dx_1}_{1} \\ &= N(t_2|\mu_1 + \mu_2, \sigma_1^2 + \sigma_2^2) \end{aligned} \tag{52}$$

Where $\stackrel{*}{=}$ holds by section Normal product. Then $P(2)$ is true.

Inductive step $P(n) \Rightarrow P(n+1)$

Let,

$$P(n) = \int \cdots \int \delta(-t + \sum_{i=1}^{n} x_i) \left(\prod_{i=1}^{n} N(x_i; \mu_i, \sigma_i^2) \right) dx_1 \dots dx_n = N(t; \sum_{i=1}^{n} \mu_i, \sum_{i=1}^{n} \sigma_i^2) \tag{53}$$

We want to prove $P(n+1)$

$$P(n+1) = \int \cdots \int \delta(-t + x_{n+1} + \sum_{i=1}^{n} x_i) \left(\prod_{i=1}^{n} N(x_i; \mu_i, \sigma_i^2) \right) N(x_{n+1}; \mu_{n+1}, \sigma_{n+1}^2) dx_1 \dots dx_n dx_{n+1} \tag{54}$$

By independence

$$P(n+1) = \int N(x_{n+1}; \mu_{n+1}, \sigma_{n+1}^2) \left(\int \cdots \int \delta(-t + x_{n+1} + \sum_{i=1}^{n} x_i) \left(\prod_{i=1}^{n} N(x_i; \mu_i, \sigma_i^2) \right) dx_1 \dots dx_n \right) dx_{n+ \tag{55}$$

By inductive hypothesis

$$P(n+1) = \int N(x_{n+1}; \mu_{n+1}, \sigma_{n+1}^2) N(t - x_{n+1}; \sum_{i=1}^{n} \mu_i, \sum_{i=1}^{n} \sigma_i^2) dx_{n+1} \tag{56}$$

By base case $P(2)$,

$$P(n+1) = N(t; \mu_{n+1} + \sum_{i=1}^{n} \mu_i, \sigma_{n+1}^2 \sum_{i=1}^{n} \sigma_i^2) dx_{n+1} \tag{57}$$

Then it holds $P(n+1)$.

References

1. Herbrich R, Minka R, Graepel T. TrueSkill: A Bayesian Skill Rating System. In: Scholkopf B, Platt J, Hoffman T, editors. Advances in Neural Information Processing Systems 19. Cambridge, MA: MIT Press; 2006. p. 569–576.

2. Kschischang FR, Frey BJ, Loeliger HA. Factor graphs and the sum-product algorithm. IEEE Transactions on information theory. 2001;47(2):498–519.
